# A comparison of three different delivery methods for achieving CRISPR/Cas9 mediated genome editing in *Cichorium intybus* L.

**DOI:** 10.3389/fpls.2023.1111110

**Published:** 2023-04-12

**Authors:** Umberto Salvagnin, Katharina Unkel, Thorben Sprink, Paul Bundock, Robert Sevenier, Milica Bogdanović, Slađana Todorović, Katarina Cankar, Johanna Christina Hakkert, Elio Schijlen, Ronald Nieuwenhuis, Maria Hingsamer, Veronika Kulmer, Michael Kernitzkyi, Dirk Bosch, Stefan Martens, Mickael Malnoy

**Affiliations:** ^1^ Fondazione Edmund Mach (FEM), Centro Ricerca e Innovazione, San Michele all’Adige, TN, Italy; ^2^ Julius Kuehn-Institute (JKI), Federal Research Centre for Cultivated Plants, Institute for Biosafety in Plant Biotechnology, Quedlinburg, Germany; ^3^ Keygene N.V., Agro Business Park 90, Wageningen, Netherlands; ^4^ Department for Plant Physiology, Institute for Biological Research “Siniša Stanković”-National Institute of Republic of Serbia, University of Belgrade, Belgrade, Serbia; ^5^ Wageningen Plant Research, Wageningen University & Research, Wageningen, Netherlands; ^6^ Joanneum Research Forschungsgesellschaft mbH, Graz, Austria

**Keywords:** chicory, genome editing, CRISPR/Cas9, RNPs, protoplasts, germacrene A synthase, socio-economic impacts, environmental impacts

## Abstract

Root chicory (*Cichorium intybus* L. var. *sativum*) is used to extract inulin, a fructose polymer used as a natural sweetener and prebiotic. However, bitter tasting sesquiterpene lactones, giving chicory its known flavour, need to be removed during inulin extraction. To avoid this extraction and associated costs, recently chicory variants with a lower sesquiterpene lactone content were created by inactivating the four copies of the germacrene A synthase gene (*CiGAS-S1, -S2, -S3, -L*) which encode the enzyme initiating bitter sesquiterpene lactone biosynthesis in chicory. In this study, different delivery methods for CRISPR/Cas9 reagents have been compared regarding their efficiency to induce mutations in the CiGAS genes, the frequency of off-target mutations as well as their environmental and economic impacts. CRISPR/Cas9 reagents were delivered by Agrobacterium-mediated stable transformation or transient delivery by plasmid or preassembled ribonucleic complexes (RNPs) using the same sgRNA. All methods used lead to a high number of INDEL mutations within the *CiGAS*-S1 and *CiGAS*-S2 genes, which match the used sgRNA perfectly; additionally, the *CiGAS*-S3 and *CiGAS*-L genes, which have a single mismatch with the sgRNA, were mutated but with a lower mutation efficiency. While using both RNPs and plasmids delivery resulted in biallelic, heterozygous or homozygous mutations, plasmid delivery resulted in 30% of unwanted integration of plasmid fragments in the genome. Plants transformed *via* Agrobacteria often showed chimerism and a mixture of *CiGAS* genotypes. This genetic mosaic becomes more diverse when plants were grown over a prolonged period. While the genotype of the on-targets varied between the transient and stable delivery methods, no off-target activity in six identified potential off-targets with two to four mismatches was found. The environmental impacts (greenhouse gas (GHG) emissions and primary energy demand) of the methods are highly dependent on their individual electricity demand. From an economic view - like for most research and development activities - employment and value-added multiplier effects are high; particularly when compared to industrial or manufacturing processes. Considering all aspects, we conclude that using RNPs is the most suitable method for genome editing in chicory since it led to a high efficiency of editing, no off-target mutations, non-transgenic plants with no risk of unwanted integration of plasmid DNA and without needed segregation of transgenes.

## 1 Introduction

Chicory (*Cichorium intybus* L.) is a horticultural plant belonging to the family Asteraceae whose cultivars are classically divided in three main groups: leaf chicory, witloof and root chicory (Raulier et al., 2016). Perhaps the most characteristic feature of the plant is its bitterness which is caused by the presence of sesquiterpene lactones, a class of compounds that most likely evolved as a defense mechanism against herbivores (Huber et al., 2016; Padilla-Gonzalez et al., 2016). All the bitter sesquiterpenes lactones (STLs) in chicory (lactucin, lactucopicrin and 8-deoxylactucin and their oxalate derivatives) belong to the class of guaianolides and ultimately derive from germacrene A (de Kraker et al., 1998; Chadwick et al., 2013). While the presence of bitter-tasting compounds is a desirable trait in leaf chicory (e.g. Italian radicchio), these compounds need to be removed during processing in root chicory (*C. intybus* var. *sativum*) because the plant is cultivated for the industrial purpose of extracting inulin, a fructose polymer used as a natural sweetener and a prebiotic (Van Laere and Van den Ende, 2002). Despite the natural variation that exists in the STLs content in the chicory germplasm (Ferioli et al., 2015), to our knowledge there is no industrial chicory variety reported having a null content of bitter tasting compounds. Hence, there is the need for the development of new varieties with a low or null content of STLs, as it would simplify the inulin purification process, making it also more sustainable.

Unfortunately, chicory is characterized by a strong sporophytic self-incompatibility, so it is very hard to obtain highly homozygous or inbred parental lines and produce new hybrids *via* classical breeding strategies (Barcaccia et al., 2016).

In this regard, New Genomic Techniques (NGTs) like CRISPR/Cas9 based genome editing have the potential to accelerate the breeding process, ensuring the preservation of elite varieties genotypes while still causing targeted genetic modifications, thus avoiding time consuming crossings (Zhang et al., 2019). In particular, the DNA-free approach (Woo et al., 2015) with the use of ribonucleoproteins (RNPs) looks promising for its potential higher acceptance from the public when compared to genetically modified organisms (Entine et al., 2021), although the public opinion seems to be very influenced by local groups of interest. Recently, the feasibility of genome editing in *C. intybus* was proved by using both protoplast cell culture and classic Agrobacterium-mediated transformation (Bernard et al., 2019; De Bruyn et al., 2020; Cankar et al., 2021). However, these reports used different varieties, different target sequences, and most importantly different delivery methods for CRISPR/Cas9 machinery (plasmid DNA, T-DNA, RNPs), making it difficult to compare the efficiencies and to understand which approach is the most suitable to be applied to chicory breeding. In this work the genes encoding germacrene A synthase (GAS) were edited using the CRISPR/Cas9 approach. The encoded GAS enzymes catalyze the first step in the STL biosynthesis, the conversion of farnesyl pyrophosphate to germacrene A (Bouwmeester et al., 2002). Germacrene A is next oxygenated by cytochrome P450 enzymes germacrene A oxidase (GAO) and costunolide synthase (COS) to form costunolide (Liu et al., 2011). Another key step to convert costunolide to the guaianolide precursor kauniolide in chicory was recently characterized, and it involves the action of three kauniolide synthases (KLS) (Cankar et al., 2022).

CiGAS gene family is an interesting target to study differences in editing characteristics of different methods, because there are four members (CiGAS-S1, -S2, -S3 and -L) that share the same intron/exon structure and have similarities but also small differences in the coding sequence (Bogdanović et al., 2019). In particular, CiGAS-S1 and -S2 share 98% sequence identity between their coding sequences, while CiGAS-S3 has a little lower identity, close to 90% (Bogdanović et al., 2019). CiGAS-L is more divergent, with 72% of identity at the aminoacidic level (Bouwmeester et al., 2002). Mutation of the CiGAS genes was described using plasmid-based and RNP-based transient delivery methods in root chicory (De Bruyn et al., 2020; Cankar et al., 2021), and both methods resulted in the successful elimination of STLs in chicory roots.

**Figure 1:**
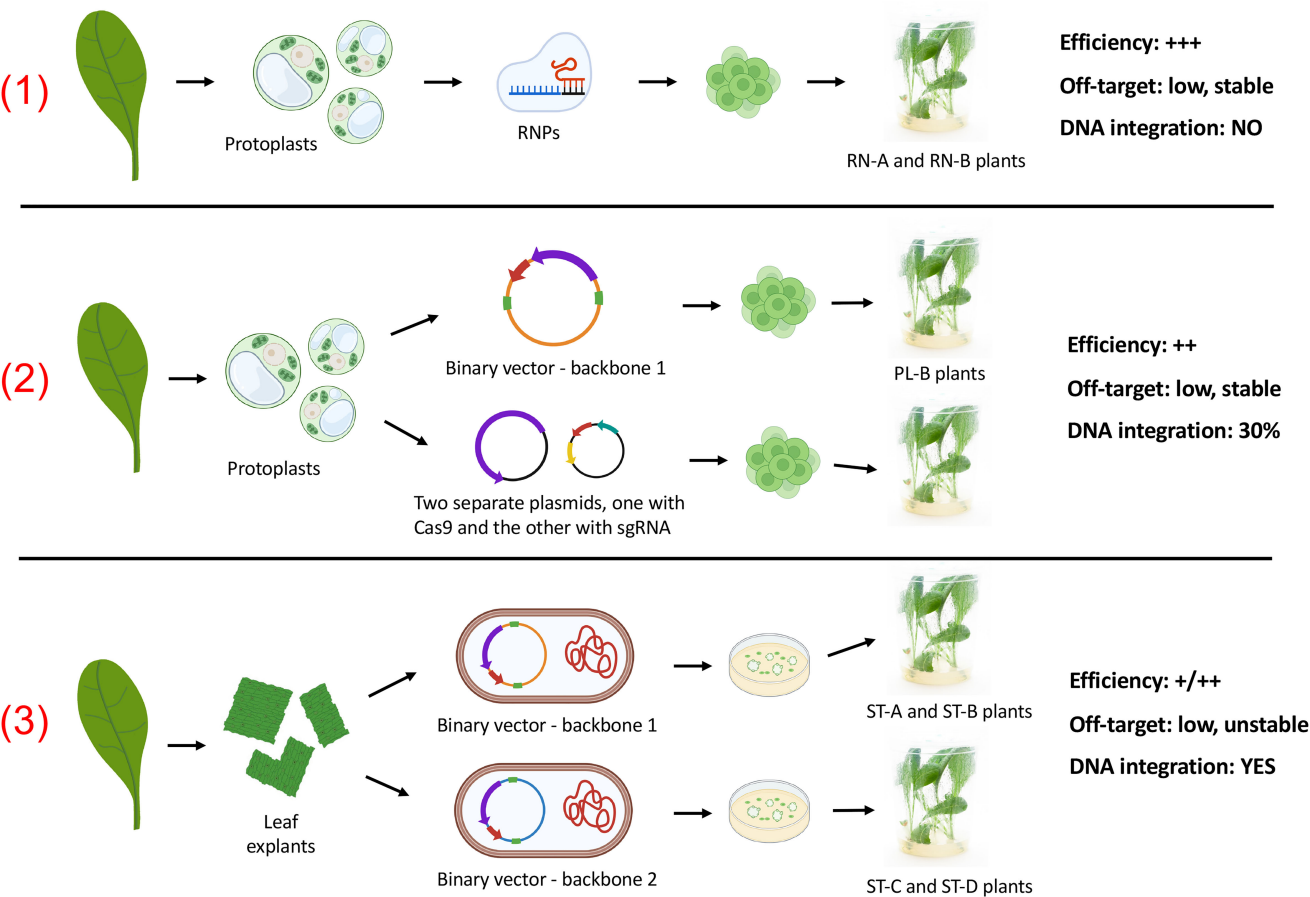
Scheme of the three CRISPR/Cas9 delivery methods as they are referred to in the main text and summary of their main outcome. The nomenclature adopted for the resulting plants is shown. Method 1: Stable integration of T-DNA containing CRISPR/Cas9 components, using two binary vectors with a different backbone. Method 2: transient expression of CRISPR/Cas9 components after protoplast transfection with one or two plasmids. Method 3: transient transfection of protoplasts directly with RNPs (DNA-free method). The purple arrow in the plasmids indicates Cas9 gene, the red arrow indicates the common guide RNA, and the green blocks indicate the Right and Left Borders (to highlight which vectors were binary and which were not).

In this work, we systematically investigated the suitability of three different delivery methods (*A. tumefaciens*, plasmid and RNPs, **Figure 1**) using the same target sequence in the same variety (clone “Orchies 37”). For this we first sequenced and *de-novo* assembled the genome of this specific clone and used plants that were generated previously by Cankar et al. (2021) and additionally generated new edited plant lines. With the aim to define the best approach specifically for chicory breeding, we considered aspects related to the delivery, the editing efficiency, but also the off-target rates and the environmental impact to produce the new gene-edited varieties. This was performed by a life cycle assessment focussing on greenhouse gas emissions and primary energy demand. Additionally, an economic comparison was done between the stable transformation and the RNP-based method, analyzing costs and broader economic impacts by applying a multi-regional input output model to quantify impacts on value added and job creation.

## 2 Materials and methods

### 2.1 Genome sequencing and assembly

1.2 g young leaves from one Chicory plant (clone “Orchies 37”) grown at Wageningen University and Research were used for high molecular weight DNA extraction using a standard CTAB-based procedure. The extracted DNA was used for Pacbio SMRT bell library preparation without any further initial DNA shearing using SMRT bell template prep kit 2.0 (Pacbio). The resulting library, with a peak insert size of approximately 41 Kb was used for subsequent template DNA polymerase complexing using Binding kit 3.0 and finally loaded for sequencing on a Pacbio Sequel system using 8 SMRT cells. For all SMRT cells data was collected using a 10-hour movie time per cell. The total Pacbio sequence data (~55 Gb) was combined with previously generated data from the same clone (~30.4 Gb). Combined data was used for a *de-novo* assembly by the Assembler Flye v2.5 running on the *in-house* HPC. The obtained assembly was polished twice, first with Racon v 1.3.3 using the reads derived from the 55Gb dataset and then using Arrow v 2.3.3.

In order to further improve the *de-novo* assembly, optical genome mapping data was applied. For this, young leaves from the same Chicory plant were harvested. These leaves were processed to isolate nuclei and finally ultra-high molecular weight DNA molecules following Bionano Prep Plant Tissue DNA Isolation Base Protocol Revision D. This DNA was used for further fluorescent labelling (DLE-1) and staining according to manufacturer’s protocol (Bionano Genomics). Labelled DNA was loaded on a single flowcell of one Saphyr chip (G1.2) and analysed on a Bionano Genomics Saphyr platform. Genome map data was used for *de-novo* assembly Using Bionano Solve v3.3 resulting in a consensus map (cmap). Finally, a hybrid assembly approach using Bionano Solve 3.4.1, Bionano Access 1.4.3 software was done to improve the polished Flye Pacbio based assembly. Therefore, the *de-novo* genome map assembly was used for scaffolding purpose and underlying genome map molecules were used for error corrections in conflict regions. Genome coverage analysis was performed by mapping raw Pacbio reads against the assembly with minimap2 v2.17-r941. Heterozygosity was identified by the presence of two peaks at approximately 20x and 40x coverage. There was an extra peak at 200x coverage that most likely contains the chloroplast genome. Next, we performed purging of haplotigs to generate a haploid assembly. The quality of this purged assembly was assessed by BUSCO analysis performed with eudicots_odb10 reference set containing a total of 2,326 BUSCO groups. By combining the sequence-based assembly and the genome mapping data a hybrid assembly “CHIC 2.0” was created consisting of 332 scaffolds with a total size of 1.76 Gb.

### 2.2 Single guide RNA design

A sgRNA was designed to introduce a frameshift mutation before the sequence coding for the “DDXXD” motif, a key feature of all terpene synthases (Chen et al., 2011) and located in Exon 4. Therefore, the four exon 4 of CiGAS-S1, -S2, -S3 and -L were aligned with ClustalW (**Supplementary Figure 6**) and the target GGTACTCTATCCCTTATGTA was chosen for being specific of CiGAS-S1 and CiGAS-S2 while having one mismatch in CiGAS-S3 and CiGAS-L, although at different positions.

### 2.3 Isolation and transfection of chicory protoplasts

Protoplast isolation, transfection and culture was performed as previously described (Yoo et al., 2007; Deryckere et al., 2012; Cankar et al., 2021) with some modifications. Briefly, *in vitro* shoot cultures of *C. intybus* var. *sativum* (clone “Orchies 37”) were maintained on MS20 medium (MS salt including vitamins (Duchefa), sucrose 20 g/L, pH 5.7) with 0.7% agar in high plastic jars at 16/8 h photoperiod under 100 μmol m^-2^ s^-1^ PPFD at 24°C. Four young leaves were harvested, rapidly sliced parallel to their long axis and placed in a Petri dish containing 13 ml of P0 liquid medium + 1% w/v Cellulase Onozuka R-10 + 0.3% w/v Macerozyme Onozuka R10. Digestion was carried out at room temperature for 16 h under gentle tilt-shaking, in the dark. The protoplasts were filtered through a 60 μm nylon sieve and then harvested by centrifugation for 4 minutes at 80 x g without brake. Protoplasts were then resuspended in 2 ml of W5 buffer (Yoo et al., 2007) then added to a tube containing 21% w/v sucrose solution: this was then centrifuged for 4 minutes at 90 x g at room temperature. Live protoplasts were then harvested from the interphase, transferred to a fresh tube, and washed with 11 ml of W5. The protoplasts were centrifuged again (4 minutes at 90 x g) and gently resuspended in MMG buffer (Yoo et al., 2007) at a density of 10^6^ protoplasts ml^-1^ (checked with a hemocytometer (Biosigma)). 20 μg of Cas9 (Thermofisher) + 20 μg of sgRNA (Thermofisher or Synthego) + 8 μl NEBuffer 3 (NEB) or alternatively 25 μg of plasmid - were mixed with 2.5 x 10^5^ protoplasts and an isovolume of PEG solution (400 g/l polyethylene glycol 4000 (Sigma-Aldrich); 0.2 M mannitol; 0.1 M CaCl_2_) was then added by gentle pipetting. The transfection was allowed to take place for 5 minutes at room temperature followed by the addition of 12 ml of WI buffer (Yoo et al., 2007). The protoplasts were harvested by centrifugation for 4 minutes at 80 x g.

### 2.4 Plant regeneration from protoplasts

For regeneration both WT and transfected protoplasts were gently resuspended in WI buffer at 0.2 x 10^6^ cells ml^-1^. An equal volume of alginate solution (1.6% w/v sodium alginate; 0.5 M mannitol) was added and gently mixed, and 1 ml of the mixture was layered on a Ca-agar (50 mM CaCl_2_; 0.4 M mannitol; 1.4% agar) plate (5 cm dish, Thermofisher), forming a disk. The alginate was allowed to polymerize for 40 minutes and the disk was then transferred to another 5 cm Petri dish containing 5 ml of MC1 medium (Deryckere et al., 2012). After 7 days of culture in the dark at 24°C the medium was replaced with 5 ml of MC2 medium (Deryckere et al., 2012) and the disk was cultured for further 14 days changing the medium another time at day 14 after the embedding. The disk was then cut into strips and transferred to a 9 cm Petri dish with solid B5 medium (Gamborg’s B5 salts including vitamins, mannitol 36 g/L, sucrose 10 g/L, glutamine 750 mg/L, Low Melting Agarose PPC (Duchefa) 0.8% (w/v), NAA 0.5 mg/L, BAP 0.5 mg/L, pH 5.75). These were incubated at 24°C in dim light (20 μmol m^-2^ s^-1^ PPFD) for two weeks to form microcalli. For each experiment approximately 200 microcalli were picked with fine tweezers and transferred to solid MS10 plates (MS salt including vitamins, sucrose 10g/L, Low Melting Agarose PPC (Duchefa) 0.8%/w/v), IAA 0.25 mg/L, BAP 0.25 mg/L, pH 5.7) and incubated at 24°C under low light (60 μmol m^-2^ s^-1^ PPFD) until green calli were formed (3 weeks). The green calli were transferred to solid MC3 medium (Deryckere et al., 2012) under full light until shoots were visible. The developing shoots (20-60 per experiment) were then moved and rooted on MS20 medium (pictures of the whole regeneration process are visible in **Supplementary Figure 5**, upper part).

### 2.5 Stable transformation

*C. intybus* clone “Orchies 37” plants were grown under sterile conditions on MS medium containing 30 g/L sucrose and 8 g/L micro agar (pH 5.8) at 25°C, with a 16 h/8 h (light/dark) photoperiod of white fluorescent light at 80 μmol m-2 s-1. *A. tumefaciens* AGL0 strain carrying the proper binary vector (**Supplementary Figures 3, 4**) was grown overnight at 28°C at 250 rpm in LB medium supplemented with 50 mg/L kanamycine, 50 mg/L rifampicin and 100 μM acetosyringone. The bacterial cells were pelleted and resuspended in MS30T medium (4.4 g/L MS medium, 30 g/L sucrose, 500 mg/L tryptone, pH5.8) supplemented with 100 μM acetosyringone at optical density (OD_600_) of 0.3. Leaf explants of approximately 0.5 cm^2^ from 4 to 6 weeks old plants were immersed for 15 minutes in the bacterial suspension, placed on co-cultivation medium (4.4 g/L MS medium, 30 g/L sucrose, 500 mg/L tryptone, 1 mg/L BAP, 0.1 mg/L IAA, 100 μM acetosyringone, 8% micro agar, pH 5.8) and incubated for 2 days at 25°C under 16/8 h (light/dark) photoperiod with white fluorescent light at 30 μmol m-2 s-1. Next, explants were rinsed with MS30T medium containing 500 mg/L cefotaxime and transferred to regeneration medium (4.4 g/L MS medium, 30 g/L sucrose, 500 mg/L tryptone, 8% micro agar, pH5.8) supplemented with 1 mg/L BAP, 0.1 mg/L IAA, 100 mg/L kanamycin, 250 mg/L cefotaxime and 50 mg/L vancomycin. Explants were incubated for 7 days at 25°C under 16/8 h (light/dark) photoperiod with white fluorescent light at 60 μmol m-2 s-1. Next, the explants were transferred to the regeneration medium containing 1 mg/L kinetin, 0.4 mg/L IAA, 100 mg/L kanamycin, 250 mg/L cefotaxime and 50 mg/L vancomycin and the medium was frequently refreshed to avoid Agrobacterium outgrowth. After 6 weeks kinetin and IAA were omitted from regeneration medium. From two months on shoots were collected and placed on rooting medium (4.4 g/L MS medium, 20 g/L sucrose, 8% micro agar, 50 mg/L kanamycin pH5.8). Pictures of the whole regeneration process are visible in **Supplementary Figure 1**, lower part.

### 2.6 Genotyping of the plants and PCR to detect the presence of Cas9 sequence

Genomic DNA was isolated from young leaves using the NucleoSpin Plant II kit (Machery-Nagel) according to the manufacturer’s instruction. The exon 4 of CiGAS-S1 and CiGAS-S2 containing the target site was amplified with specific primers (**Supplementary Table 2**) and overhang Illumina adapters to generate the Illumina library amplicons, which were sequenced on an Illumina MiSeq (PE300) platform (MiSeq ControlSoftware 2.0.5 and Real-Time Analysis Software 1.16.18) as reported by (Quail et al., 2012). The CRISPResso2 pipeline (https://crispresso.pinellolab.partners.org/submission; (Clement et al., 2019)) was used to process the raw paired-end reads and to visualize the mutations profiles.

To detect T-DNA integration in the case of stable transformation, or plasmid integration in the case of transient plasmid delivery, a PCR was performed using genomic DNA as template (100 ng) and the primer pair Cas9wt for (CTTCAGAAAGGACTTCCAATTC) and Cas9wt rev (ATGATCAAGTCCTTCTTCACTT), using PCRBIO Taq Mix Red (PcrBiosystems) according to manufacturer’s instructions. A single specific amplicon of 693 bp was obtained in the case of positive signal.

### 2.7 Off-target analysis

Sequencing primers have been created using Primer3web (version 4.1.0.) (Untergasser et al., 2012), and the reverse primers have been tagged by five nucleotides, in order to pool the amplicons coming from different plants in the flow cell and separate them later in the analysis. Possible hybridisation between tag-combinations within the sample pools has been checked using the open-source software package “edittag” (Faircloth and Glenn, 2012). The short *Ci*GAS genes S1 and S2 have been amplified by nested PCR or from genomic DNA directly, whereas S3 and the long *Ci*GAS-L gene were amplified directly *via* PCR. Amplification took place in a mixture containing 0.5 U Phusion™ High-Fidelity DNA Polymerase; 0.2 µM dNTPs; 0.4 µM forward and tagged reverse primer in 10 µL 5x High-Fidelity buffer with added ddH_2_O up to 50 µL. Initial heating was performed at 98°C for 30 sec., followed by dehybridisation at 98°C for 30 sec., annealing at 60°C for 30 sec., and elongation at 72°C for 30 sec., all but the initial heating was repeated 30 times, followed by a final elongation at 72°C for four minutes. The PCR mixture was kept at 4°C until verification of the correct amplification took place on a 2% TAE agarose gel, containing 0.005% Midori green Advance (NIPPON Genetics EUROPE GmbH). Then 45 µL of the PCR mixture was purified by column (Thermo Fisher Scientfic) and its DNA content measured *via* NanoDrop (Thermo Fisher Scientific) and normalized to 20 ng/µL. The samples have been pooled by four plants each by combining 10 µL of four single samples and send to Genewiz/Azenta (Leipzig, Germany) for Amplicon-EZ (150-500 bp). The raw data was analyzed using the Galaxy JKI server. Paired reads were adjusted to the correct orientation using the forward primer. The quality trimming was done by Trim Galore! (Galaxy Version 0.6.3.), with the Phred Quality Score set between 30 to 35 and the minimum length set to 50. After trimming, the data set was split according to their tags using the Sabre tool (version 1.000). The tool usearch (v11.0.667_i86linux32) (Edgar et al., 2011) was then used to merge the two read pairs into one sequence. When merging forward and reverse read, up to five mismatches were allowed due to large overlaps between the reads. The merged datasets have been transformed into FASTA files, dereplicated and counted using vsearch (version 2.8.3) (Rognes et al., 2016). Then, only one representant of the identical sequence was mapped against the wild type sequences *via* BWA-MEM (Li and Durbin, 2010). The result was sorted according to the number of identical sequences. The output was aligned in the CLC Main Workbench 22.0 (QIAGEN Aarhus A/S). Spiked fastq files including six artificial mutated reads have been use as a positive control to verify the thoroughness of the workflow for finding off-target events. Additionally, sequences showing a variant in comparison to the wild type that were supported by at least 20 reads have been examined. For this purpose, individual Phred Quality Scores of the merged reads were collected and visualized as boxplot and median per position. If a drop coincides with a variant position, this variant was flagged as questionable.

### 2.8 Environmental assessment

Life Cycle assessment (LCA) defined in the International Standards ISO 14040 is a method to compile and assess the input and output flows as well as the potential environmental impacts of a product system during the various stages of its life cycle. The stages include extraction of raw materials, manufacturing, distribution, product use, recycling and final disposal (from cradle to grave/gate) (ISO 14040). A “cradle to gate” LCA was applied, and the impact focused on was greenhouse gas (GHG) emissions and primary energy demand. The functional unit is one experiment consisting of three cycles to gain one prototype of the Chicory variant with the desired characteristics. For the assessment the operational phase with all the needed inputs of the molecular breeding steps is included in the system boundary, the construction and end of life of the laboratories or machinery are not included. For the assessment of the contribution of the GHG emissions, the global warming potential on 100-year time horizon (GWP 100) was used. The GHG - CO_2_, CH_4_, N_2_O - were expressed in terms of equivalent amount of CO_2_ (CO_2_-eq). Therefore, the CO_2_-eq factors are taken from (Myhre et al., 2013) using the factors including climate carbon feedback. Direct and indirect emissions are included in the assessment. The cumulated primary energy demand includes the total energy demand (fossil, renewable and other primary energy demand) of all process steps of the life cycle analysis. Two types of data are used in the LCA calculation - namely foreground and background data. Foreground data was mainly based on information collected from the laboratory work (mainly materials and energy demand for the molecular breeding technologies), while background data for materials, fuels and transport was mainly gathered from the database Ecoinvent 3.7.1 (Wernet et al., 2016). Information on the electricity mix for the EU28 mix is drawn from European Commission (2020).

### 2.9 Economic assessment

Multi-regional input-output (MRIO) analysis is conducted to quantify costs and broader economic impacts of the breeding methods. MRIO analysis is based on Leontief (Leontief, 1970) and relies on a set of linear equations. Starting from the basic balance of the input-output table, a series of equations can be deduced for the economic impact calculations. The total output can be expressed as Eq. 1 (in order to enhance readability and clarity for the reader we abstract from a sector disaggregation):


(1) $$\left[\begin{array}{c} X_{1}\\ X_{2}\\ \vdots \\ X_{R} \end{array}\right]=\left[\begin{array}{cccc} A_{11} & A_{12} & \cdots & A_{1R}\\ A_{21} & A_{21} & \cdots & A_{2R}\\ \vdots & \vdots & \ddots & \vdots \\ A_{R1} & A_{R2} & \cdots & A_{RR} \end{array}\right]*\left[\begin{array}{c} X_{1}\\ X_{2}\\ \vdots \\ X_{R} \end{array}\right]+\left[\begin{array}{c} Y_{1}\\ Y_{2}\\ \vdots \\ Y_{R} \end{array}\right]$$

where *X_i_* denotes the column vector of total output by economic sector in country *i* (*i*,*j* ∈ *R)*, *A* is a coefficient matrix describing input per output ratios in the production of these sectors with *A_ij_* denoting inputs from sectors in country *i* required to produce one unit of output from each sector in country *j*. *Y_i_* is a column vector of total final demand for the output of country *i*. This equation can be transformed into *X*=(*I*-*A*)^-1^ *Y*, where (*I-A*)^-1^ describes the inverse Leontief matrix, which captures all the direct and indirect links between the different economic sectors as well as regions and thus enumerates the total impacts across global supply chains. The Leontief matrix can then be extended by a row-vector of economic, social and environmental coefficients *q* (so-called satellite accounts) in order to illustrate how these indicators are distributed over sectors and countries. Thereby *q*=[*q* ^1^,*q* ^2^, ,*q^R^*] is a row vector of the respective coefficients by sector and region denoting the physical (e.g. number of jobs, land in m², water in m³) or monetary (labor and value-added in USD) units per unit of output. This row vector of coefficients times the total output needed to meet any final demand yields embodied economic, social and environmental indicators, Q, in final demand (*Q*=*q* * (*I*-*A*)^-1^ *Y*; *Q* is a R x R matrix) (Gasim, 2015). The MRIO model is calibrated to the EXIOBASE data (Wood et al., 2015; Stadler et al., 2018) of 2019. The model comprises 163 industries and covers 44 countries and five rest of the world regions by continents. Multipliers were defined as values that quantify the economic impacts derived from a perturbation on the system. These include the direct consequence caused by the initial effects as well as the indirect ripples of the total effects on the economy (Miller and Blair, 2009). The multiplier effect was calculated as the ratio between the total economic impact estimated and the amount of money directly spent in the development of both methods in terms of goods and services.

## 3 Results

**Figure 2:**
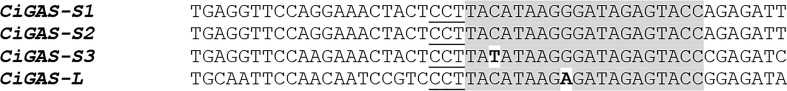
Alignments of the genomic sequences of the four CiGAS genes at the target site in exon 4. The target region of the sgRNA is highlighted in grey. The PAM is underlined, and the mismatches on CiGAS-S3 and CiGAS-L are in bold.

The target sequence to design the sgRNA common to all three delivery methods was intentionally chosen with perfect match in CiGAS-S1 and CiGAS-S2 and one mismatch in CiGAS-S3 and CiGAS-L (**Figure 2**): in this way, despite their role in germacrene A biosynthesis, CiGAS-S3 and CiGAS-L can also be considered as off-targets due to the singe mismatch. Notably, the position of the single mismatch differs in the two guides, being very close to PAM in CiGAS-S3 and in the middle of the sequence in CiGAS-L.

### 3.1 Genome assembly

In total 86 Gigabases of Pacbio long read sequencing data was produced and used for initial assembly, polishing and purging. The resulting purged assembly encompasses a total size of approximately 1249 Megabases consisting of 8773 contigs, with a N50 contig size of 469 Kb and L50 of 741 fragments. Additional purged haplotigs contained 303 Mb spanning sequences consisting of 8340 contigs. BUSCO analysis of the assembly before purging showed 94.7% BUSCO score for the initial assembly, consisting of 1418 complete single copy BUSCO and 785 complete and duplicated BUSCOs. In contrast, the purged assembly showed 94% BUSCO score, consisting of 1,823 complete single copy BUSCO and 364 complete and duplicated BUSCOs. The purging of the assembly significantly reduced the assembly size as well the BUSCO duplication rates of the initial assembly, but not the total BUSCO score suggesting. This, together with the comparison to the public *C. intybus* assembly GCA_023525715.1 (**Supplementary Figure 2**) suggest that the purged assembly is a good representation of the haploid genome of Chicory clone “Orchies 37”. *De-novo* assembly of the genome mapping resulted in a total assembly consensus map of approximately 2.5 Gb representing the diploid genome. The subsequent hybrid and final assembly CHIC v 2.0 consisting of 332 scaffolds with a N50 length of 9.81 Mb and a total size of 1.76 Gb. All statistics of the different genome assembly steps and final genome assembly are provided in supplementary data (**Supplementary Table 4** and **Supplementary Figures 1, 2**).

### 3.2 Method1: transient RNPs delivery and plant regeneration

For the DNA-free method, two types of chemically synthetized sgRNA molecules were used: 1) normal and 2) with both ends containing three phosphorothioate linkages and 2’-O-methyl RNA modifications. The last type is referred to as protected sgRNA and it is known to resist to endogenous nucleases, thus having the potential to increase its lifetime in the plant cell and its editing efficiency (O’Reilly et al., 2019; Allen et al., 2021). The mutation efficiency was first determined in a transient assay in a protoplast population, harvesting the genomic DNA 48 hours after transfection with 20 μg of Cas9 and sgRNA and then using amplicons on CiGAS-S1 and S2 genes for Illumina sequencing. The percentage of mutated reads on the total was of 52% for CiGAS-S1 and 54% for CiGAS-S2 (**Supplementary Figure 7**) with the normal sgRNA, and 38% for both genes with the modified version. In a parallel experiment, protoplasts were not sacrificed for the sequencing and were cultivated *in vitro* until plants were regenerated: these plants were genotyped as well by deep sequencing of the CiGAS genes. In total, 31 plants from the transformation with the normal sgRNA and 18 plants from the transformation with the modified guide were analysed (**Table 1**). It is important to note that since Chicory is a diploid species, in case of a monoallelic mutation (indel) it is expected that on the total amount of the Illumina reads, half is wild type, and the other half is coherently mutated. In the case of biallelic mutants, no wild type reads should be observed, with a unique mutation profile in the case of homozygosity or two equally represented in the case of heterozygosity. The absence of other genotypes in the sequenced sample indicates that the plant is not chimeric and that it derived from a single cell: indeed, this was the case for nearly all the plants generated by the RNP approach, and the deviating sequence reads with a very low frequency (i.e. less than 0.01%) of alternative genotypes could be explained by technical sequencing errors. Overall, the frequency of mutated alleles in CiGAS-S1 and -S2 which perfectly match with the guide RNA was 68% in the case of modified sgRNA and 92% in the case of the standard guide RNA, with 42% and 49% of true biallelic or monoallelic loss-of-function mutants (knockouts), respectively. In the case of CiGAS-S3 and CIGAS-L (only one mismatch with the guide) the frequency was lower compared to CiGAS-S1 and CiGAS-S2, with 47% of edited alleles (22% loss-of-function) in the case of the standard guide, and 43% of edited alleles (31% loss-of-function) in the case of the modified one. The position of the mismatch differed between CiGAS-S3 and -L (**Supplementary Figure 1**): surprisingly, the highest editing rate (87% of the alleles with standard guide, 66% with modified) was obtained in CiGAS-S3 where the mismatch is in the seed region, while in CiGAS-L, where the mismatch is in the middle of the target sequence, only 6.5% of the alleles were edited (21% with modified guide RNA).

**Table 1:** CiGAS genotype of the plants regenerated from protoplasts transfected with RNPs.

Plant #	Modified sgRNA	CiGAS-S1	CiGAS-S2	CiGAS-S3	CiGAS-L
RN-A1	Yes	WT/WT	WT/WT	WT/WT	WT/WT
RN-A2	Yes	WT/WT	WT/WT	WT/WT	WT/WT
RN-A3	Yes	WT/WT	WT/WT	WT/WT	WT/WT
RN-A4	Yes	-5/-11	-9/-9	-7/ins.	WT/WT
RN-A5	Yes	-12/-9	WT/WT	WT/WT	WT/WT
RN-A6	Yes	-19/-4	WT/WT	-4/WT	WT/WT
RN-A7	Yes	-4/-17	-11/-11	-2/-7	WT/WT
RN-A8	Yes	-14/-7	-13/-10	-2/WT	WT/WT
RN-A9	Yes	-9/-9	-10/WT	-4/-4	WT/WT
RN-A10	Yes	-3/-5	-6/-3	-7/WT	WT/WT
RN-A11	Yes	-7/-7	-5/-5	-2/-2	WT/WT
RN-A12	Yes	-4/-7	+1/-7	-7/-16	WT/WT
RN-A13	Yes	-11/-11	-12/-6	-7/-9	WT/WT
RN-A14	Yes	-7/-7	-9/-7	-3/-3	-11/WT
RN-A15	Yes	WT/-16	WT/WT	WT/-5	WT/WT
RN-A16	Yes	-2/-11	-2/-11	-7/-9	-7/-16
RN-A17	Yes	-6/-5	-10/-6	-11/+1	-8/-9
RN-A18	Yes	-12/-12	-7/-7	-18/-11	-7/-6
RN-B1	No	-3/-5	-16	-9	WT/WT
RN-B2	No	-3/-9	-6/-7	-11/-13	WT/WT
RN-B3	No	-3/-9	-6/-7	-11/-13	WT/WT
RN-B4	No	-3/-5	-16	-9	WT/WT
RN-B5	No	-3	+1	-3/-5	WT/WT
RN-B6	No	-11	-4/-1	+1	WT/WT
RN-B7	No	-3/-9	-6/-7	-11/-13	WT/WT
RN-B8	No	-3/-9	-6/-7	-11/-13	WT/WT
RN-B9	No	-3/-5	-16	-9	WT/WT
RN-B10	No	-3/-5	-16	-9	WT/WT
RN-B11	No	-3/-5	-16	-9	WT/WT
RN-B12	No	-16/-3	-11/-6	+1/WT	WT/WT
RN-B13	No	-16/-3	-11/-6	+1/WT	WT/WT
RN-B14	No	-11/-6	-14/-7	-9/WT	WT/WT
RN-B15	No	-11/-6	-14/-7	-9/WT	WT/WT
RN-B16	No	-9/-3	-6/-7	-13/-11	WT/WT
RN-B17	No	-9/-3	-6/-7	-13/-11	WT/WT
RN-B18	No	-3	-16/-7	-3	-9/WT
RN-B19	No	-5/-3	-16	-9	WT/WT
RN-B20	No	-5/-3	-16	-9	WT/WT
RN-B21	No	WT/WT	WT/WT	-8	-7/WT
RN-B22	No	-9	-4/-11	-11/-16	WT/WT
RN-B23	No	+1/-3	-4/-4	-6/-3	-7/WT
RN-B24	No	-3	+1	-3/-5	WT/WT
RN-B25	No	WT/WT	WT/WT	WT/WT	WT/WT
RN-B26	No	-7/-9	-2	-6	WT/WT
RN-B27	No	-9/-7	-9/-1	-14/-15	WT/WT
RN-B28	No	WT/WT	-3	WT/WT	-9/WT
RN-B29	No	-12/-3	-3	-3	WT/WT
RN-B30	No	-4/-11	-16/+1	-3/-26	WT/WT
RN-B31	No	-4/-5	-5/-3	-2/-4	WT/WT

Light grey colour indicates cases where no editing occurred. Medium grey colour indicates out of frame biallelic or homozygous mutations resulting in inactivation of the enzyme. No colour indicates either plants with in-frame deletions or plants in which the presence of one WT allele was detected.

### 3.3 Method 2: transient plasmid delivery and plant regeneration

For the DNA-based transient expression, two systems were used where sgRNA and Cas9 cassettes were in the same construct (single plasmid approach) or expressed from two independent plasmids (double plasmid approach). In the first strategy the same binary vector used in Method 3 for *Agrobacterium*-mediated transformation was exploited: the plasmid (**Supplementary Figure 4**) was 16.7 kb in size and contained in the T-DNA part the sgRNA under the control of an extended *A. thaliana* U6 promoter and a SpCas9 ORF with N- and C-terminal NLS (SV40) under the control of *A. thaliana* ubiquitin 10 promoter. In the second strategy, three guides hybridising to the same target sequence as the guide used in Method 1 was used, which was adjusted to have no mismatches to the different CiGAS genes. The guides were expressed under the control of the *A. thaliana* minimal U6 promoter and cloned into pMK-RQ vector (GeneArt). For expression of the *Sp*Cas9 protein, a coding sequence codon-optimized for *A. thaliana* with a C terminus NLS was used under the control of the parsley constitutive ubiquitin promoter. Protoplasts transfection efficiency was assessed in a transient assay in a protoplast population, using 20 μg of a plasmid bearing a fluorescent reporter cassette: fluorescent signal was detected in 58 ± 6% of the cells after 24 hours. As with method 1, the editing efficiency was evaluated through deep Illumina sequencing on CiGAS amplicons: in total, we analyzed 7 plants from the transient transformation with the binary vector and 9 plants from the transformation with the double plasmid system (**Table 2**). Overall, the frequency of mutated alleles in CiGAS-S1 and -S2 was 53% in the case of the single plasmid and 50% in the case of the double plasmid, with 14% and 39% of loss-of-function alleles, respectively. The single plasmid strategy in CiGAS-S3 and -L mirrored the RNP approach: both targets had the same mismatch in the same position, and in fact the editing results turned out to be similar, although at lower frequencies compared to RNPs. In particular, CiGAS-S3 maintained a high frequency of mutation (78% with 50% of loss-of-function) while CiGAS-L showed almost no editing (7%). The case of the double plasmid delivery (where the three guides were used with no mismatch in any of the genes) gave a very consistent output, with an average of 60% of edited alleles in each locus, whose majority were loss-of-function. An event known to occur when performing a transient plasmid delivery is the potential integration of fragments of plasmid DNA into the genome of the plant, which then results being transgenic and thus with heavier regulatory implications in many countries (Entine et al., 2021). Although the frequency might vary between species and it is generally considered to be low, however it was recently reported in Chicory to occur relatively frequently (Bernard et al., 2019). To assess the level of plasmid integration in the plants we performed a PCR on the SpCas9 ORF, which was present in 30% of the lines, proving that significant foreign DNA integration had indeed occurred.

**Table 2:** CiGAS genotype of the plants regenerated from protoplasts transfected with plasmid.

Plant #	plasmid	CiGAS-S1	CiGAS-S2	CiGAS-S3	CiGAS-L
PL-A1	Double	WT/WT	WT/WT	WT/WT	WT/WT
PL-A2	Double	WT/WT	WT/WT	WT/WT	WT/WT
PL-A3	Double	WT/WT	WT/WT	WT/WT	WT/WT
PL-A4	Double	WT/WT	WT/WT	WT/WT	WT/WT
PL-A5	Double	-9/-12	WT/WT	-2/-9	-6/-6
PL-A6	Double	-1/-1	-4/-4	-2/-11	-1/-5
PL-A7	Double	-3/-3	-2/-2	-4/-4	-5/-5
PL-A8	Double	-13/-13	-11/-11	-7/-7	-2/-2
PL-A9	Double	-7/-8	-4/-4	-9/-7	-4/-5
PL-B1	Single	-20/WT	-9/-9	-10/-10	WT/WT
PL-B2	Single	WT/WT	WT/WT	-4	WT/WT
PL-B3	Single	-9	-7/complex	-5/-8	WT/WT
PL-B4	Single	WT/-9	-8/WT	WT/-5	WT/WT
PL-B5	Single	WT/WT	WT/WT	-13/WT	WT/WT
PL-B6	Single	WT/-14	-8/WT	-9/WT	WT/-8
PL-B7	Single	-9/-5	-7/-4	-9/-4	WT/WT

Light grey colour indicates cases where no editing occurred. Medium grey colour indicates out of frame biallelic or homozygous mutations resulting in inactivation of the enzyme. No colour indicates either plants with in-frame deletions or plants in which the presence of one WT allele was detected.

### 3.4 Method 3: Stable T-DNA integration and plant regeneration from leaf explants

**Figure 3:**
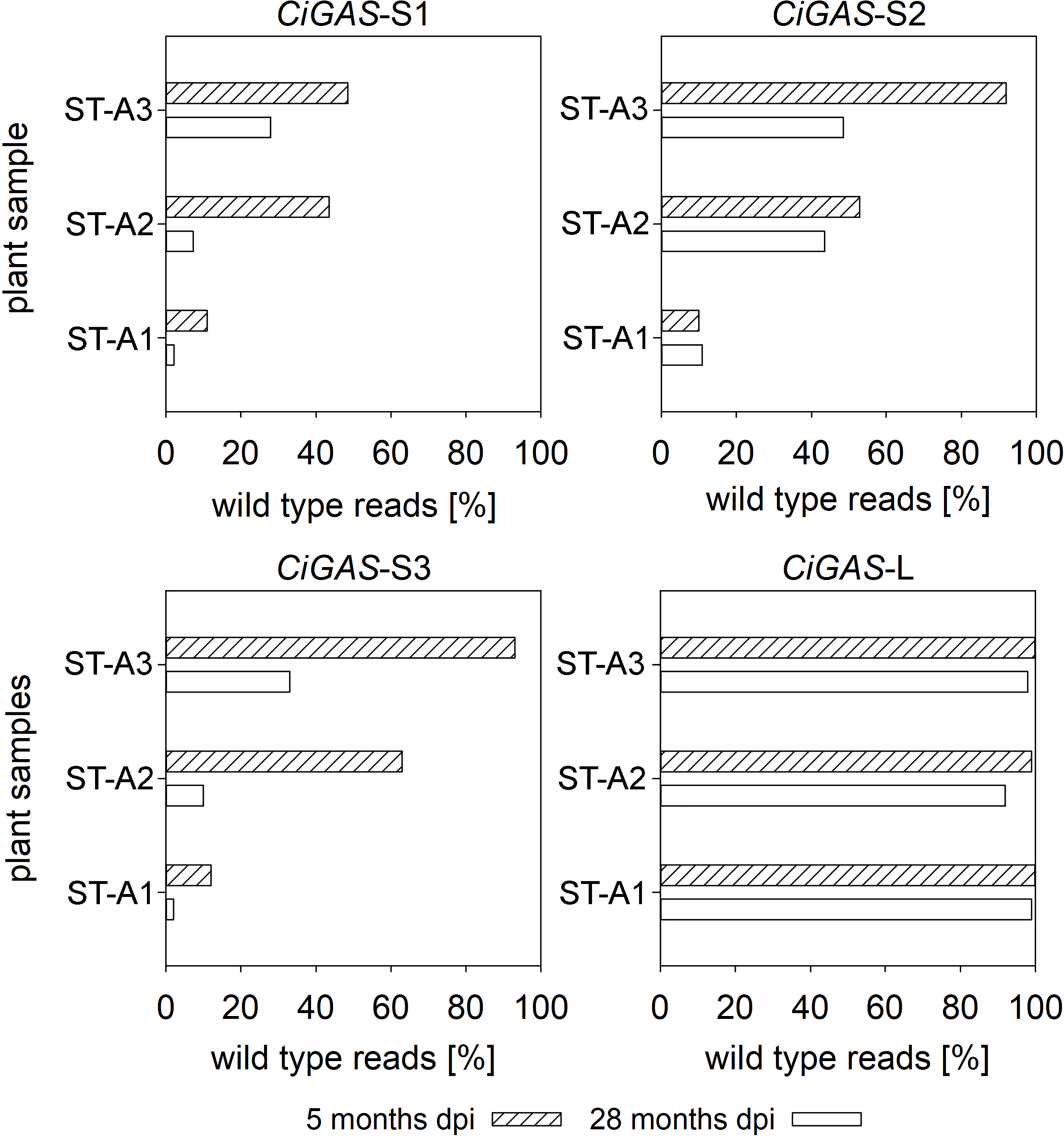
Change of wild type reads in transgenic chicory plants over two years. DNA was isolated five months and 28 months after transformation. All three plants show a decrease in their percentage of wild type sequences in NGS approach.

For the stable integration, two different binary vector backbones were used: one derived from pICSL4723 (Weber et al., 2011) was called backbone 1 (**Supplementary Figure 3**), and another one derived from pPZP (Karimi et al., 2002) called backbone 2 (**Supplementary Figure 4**). Four independent transformations were performed, and a fraction of all the regenerants (Cas9-positive by PCR) from all four regeneration experiments were screened by deep amplicon sequencing of all CiGAS genes as in the previous methods (**Table 3**). Chimerism was common in all of the different transformations, indicated by the fact that more than two alleles were detectable for each locus in most of the cases. When only two alleles were present, they were rarely in a 50:50 ratio. Since transgenic lines have stably integrated CAS9 gene, ongoing Cas9 activity is to be expected during the whole lifetime of the plants, possibly causing a change in the mutation profile of *CiGAS* genes during plant development. Therefore, three independent transgenic lines were selected and sampled at two time points: 5 months after the transformation and 23 months later after continuous *in vitro* propagation. The amount of wild type reads in the sequencing detectable at the beginning decreased (**Figure 3**) and the amount of new occurring indel mutations increased in all plants indeed indicating that the genes coding for Cas9 and the sgRNA had not been silenced in the timeframe considered, producing active CRISPR complexes that could bind to the targets and achieve new mutations.

**Table 3:** CiGAS genotypes of transgenic plants 5 months after agrobacterium-mediated transformation.

Plant #	Trasf.	Plasmid	CiGAS-S1	CiGAS-S2	CiGAS-S3	CiGAS-L
ST-A1	1	Backbone1	+1/-214/-6	-21/-7/WT	-4/+1/WT	WT
ST-A2	1	Backbone1	WT/+1/-7	WT/-8/-6	WT/+1/-9	WT
ST-A3	1	Backbone1	WT/-5	WT	WT	WT
ST-B1	2	Backbone1	-7/-6/+1/*	-14/+1/-3	-14/+1/WT	WT
ST-B2	2	Backbone1	+1/WT	-16/-11/+1	-16/-7/-4	WT
ST-B3	2	Backbone1	-4/-10/+1	+1/-4	WT/+1/-2	WT
ST-B4	2	Backbone1	-20/-4/+1	+6/+17	-2/+1	WT
ST-B5	2	Backbone1	WT/-11/-5	-9/-88/WT	-21/WT/-11	WT
ST-B6	2	Backbone1	+1/-4	-2/+7/+1	WT/-6/+1	WT
ST-B7	2	Backbone1	-20/-4	-3/-10	-6/-20	WT
ST-B8	2	Backbone1	-11/-15/+1	+1/-6/-7	-52/-7/-10	WT
ST-B9	2	Backbone1	-7/+1	-6/-9	-2/WT/+1	WT
ST-B10	2	Backbone1	+1/-1	-9/+1	+1/-2/-6	WT
ST-C1	3	Backbone2	WT	WT	WT	WT
ST-C2	3	Backbone2	-7/-5	-9/-7/-6	+1/-11	WT
ST-C3	3	Backbone2	-7/-5	-9/-6/+1	-11/+1	WT
ST-C4	3	Backbone2	-7/-5	-9/WT/-6	+1/-11	WT
ST-C5	3	Backbone2	-7/WT/-6	-6/-2	WT/-6/+1	WT
ST-C6	3	Backbone2	-7/-6	-11/-2/WT	WT/+1/-9	WT
ST-C7	3	Backbone2	WT	WT	WT	WT
ST-C8	3	Backbone2	-27/WT	-27/WT	WT/-6	WT
ST-C9	3	Backbone2	WT	WT/-2	WT	WT
ST-C10	3	Backbone2	-4/WT/-11	-6/+1	WT/+1	WT
ST-C11	3	Backbone2	-4/WT/-5	-6/+1	WT/+1	WT
ST-D1	4	Backbone1	-7/WT/+1	-6/-7/WT	WT/-2/+1	WT
ST-D2	4	Backbone1	-6/WT/-7	WT/-26/-11	WT/-5/+1	WT
ST-D3	4	Backbone1	+1/WT/-7	-6/+1/WT	WT/+1/-9	WT
ST-D4	4	Backbone1	WT/+1/-9	WT/+1/-6	-11/WT/-3	WT
ST-D5	4	Backbone1	WT/+1/-3	-2/WT/+1	-11/WT/-6	WT
ST-D6	4	Backbone1	WT/-4/+1	WT/+1/-4	WT/-9/-7	WT

The mutations are shown in decreasing order or reads count. For visual purpose, the threshold for a genotype to be displayed in the table was set to 3% of total reads, with the only exception of the locus CiGAS-S1 of line ST-B1 (indicated with *). Light grey colour indicates cases where no editing occurred. Medium grey colour indicates complete loss-of-function. No colour indicates editing which cannot be considered for sure loss-of-function because the indel occurred in multiples of 3bp, or because WT reads are still detectable.

### 3.5 Off-target analysis

Even though the mutation frequency in *CiGAS*-S3 and -L was lower compared to *CiGAS*-S1 and CiGAS-S2, genome editing in these genes was detected despite single mismatches between the target sequence and the sgRNA. Therefore the improved genome CHIC2.0 was screened by the prediction algorithms CCTop (Stemmer et al., 2015) and CRISPOR (Haeussler et al., 2016) to identify possible off-target sites. While other online tools are available, CCTop and CRISPOR allow working with unpublished genome data on local server. A systematic review found possible off-target activity with four or less mismatches between sgRNA and genomic sequences (Modrzejewski et al., 2020). The screening for off-targets with up to four mismatches between the sgRNA used to mutate the four *Ci*GAS genes and the CHIC2.0 genome identified 18 potential off-targets (**Supplementary Table 1**). Of those off-targets, two mismatched the sgRNA in two bases, one in three bases and the other putative off-targets sides showed four mismatches to the sgRNA. Those sequences have been annotated by GeMoMa (Gene Model Mapper) (Keilwagen et al., 2016) using RNA-Seq data from Chicory as well as the annotated genomes from *Arabidopsis thaliana* L., soybean (*Glycine max* (L.) Merr.*)*, sunflower (*Helianthus annus* L.) and lettuce (*Lactuca sativa* L.) to find homologies for gene prediction. With high variations within intragenic regions (Intrieri et al., 2007), the off-target search focused on deep analysis of five putative off-targets found in genomic regions, which are more conserved and could result in changes of the plants’ phenotype. Additionally, one putative off-target side showed only two mismatches to the target sequence and was therefore included in the deep analysis. Possible off-target activity within those six regions were analysed by deep sequencing in 13 plants treated with RNPs, nine plants transfected *via* plasmid and 18 plants stably transformed by Agrobacterium. In none of these 40 plant lines mutations were detected in these potential off-target sites (**Table 4**). Even the prolonged exposure over two years to continuously expressed CRISPR/Cas9 within the stably transformed plants showed no off-targets mutations. To assess whether any of the 18 identified potential off-targets is prone to being mutated by the CRISPR/Cas9 delivery, one of the RNP-transfected plants with high mutation efficiency of the target site was deep sequenced, as well as two stably transformed plants. Also here, no difference between transformed plants and wild type controls could be detected.

**Table 4:** Total number of plants analysed for at least six possible off-targets.

Method	Number of plants	Modified sgRNA	mutated off-targets	Transfection/Transformation efficiency	Editing efficiency
RNP	13	no/yes	0*	n.d.***	80%
Plasmid	9	No	0	n.d.***	51.5%
Stable	18	No	0*	60%****	90%**

*one RNP treated and two transgenic plants were checked in all 18 possible off-target sides.

**being all the plants chimeric, this was calculated as percentage of loci where edited reads could be detected.

***in the case of transient delivery, the transfection efficiency was not determined, but it is at least equal to the editing efficiency.

****calculated as the number of Cas9 positive plants (detected by PCR) on the total number of regenerants.

Given are the methods of transformation and whether modified or unmodified sgRNA was used. For comparison, also the overall efficiencies of editing, transformation and transfection are reported.

### 3.6 Environmental impacts of the molecular breeding approaches

**Figure 4:**
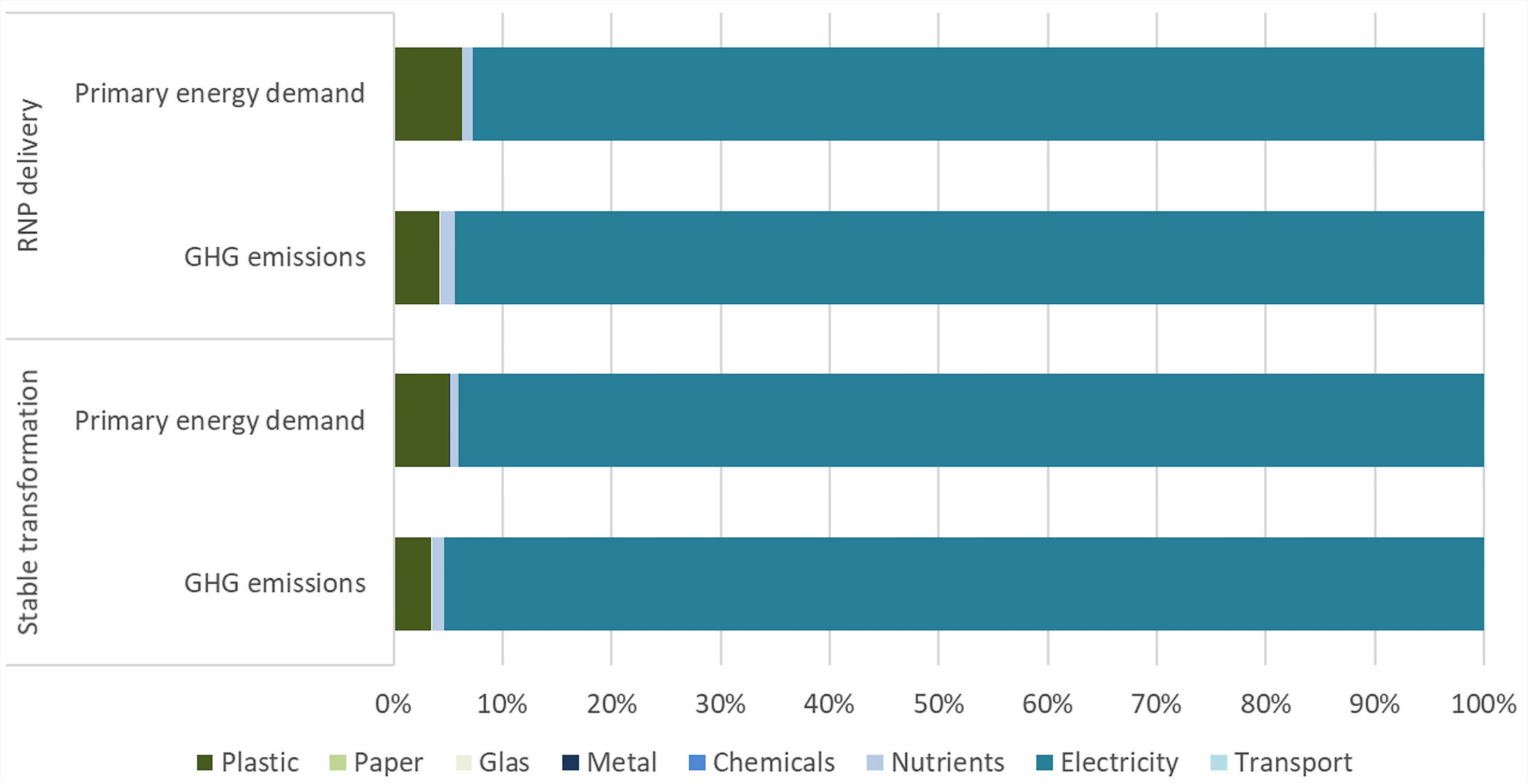
Shares of environmental impacts by auxiliaries for stable transformation and RNP delivery.

For the environmental assessment the two molecular breeding technologies showing the largest differences in the frequency of introduction of loss-of-function mutations were used: the RNPs delivery (Method 1) and the stable transformation (Method 3) were analyzed and compared. To assess the environmental impacts the method of an attributional “Life Cycle Assessment (LCA)” was applied according to ISO 14040 considering one experiment with the need of three cycles to gain one prototype of the Chicory variant with the desired genotype to put on the market. All the material and energy demands of the molecular breeding technologies were included in the assessment and translated to GHG emissions and primary energy demand. To cover the electricity demand, the GHG intensity of a European electricity mix was applied. For the molecular breeding technologies an average electricity demand of lighting of 2.56 kWh per day was assumed. In general, only minor differences between the two technologies were found when comparing the greenhouse gas (GHG) emission (**Figure 4**) and the cumulated primary energy demand. The GHG emissions of the stable transformation method is estimated between 487 and 703 kg CO_2_eq per experiment (from here on/e), and for the RNP delivery method between 492 and 710 kg CO_2_ eq/e. If we are comparing these values with GHG emissions of a passenger car, we could drive approx. 2,200 to 3,100 passenger car-km/e, assuming 226 g CO_2_eq/passenger car-km. If renewable energy would be used to cover the electricity demand the GHG emissions can be reduced significantly by 84% to 88% to approx. 59 to 105 kg CO_2_eq/e in the case of stable transformation and to approx. 65 to 112 kg CO_2_eq/e in the case of RNP delivery. The cumulated primary energy demand of stable transformation is estimated between approx. 2,900 and 4,250 kWh/e, of the RNP delivery between approx. 2,940 and 4,300 kWh/e assuming an EU 28 electricity mix to cover the electricity demand. If renewable energy is used to cover the electricity demand the cumulated primary energy demand can be reduced significantly by 41% to 44%. The electricity demand accounts for the biggest share of contributions to the GHG emissions and the primary energy demand followed by the contribution of plastic (**Figure 4**). In fact, with the EU28 electricity mix approx. 95% of the GHG emissions derives from the electricity demand. This value can be reduced to about 66% in the case of stable transformation and to 62% in the case of RNP delivery when using renewable electricity. The share of electricity to the primary energy demand can be lowered from approx. 94% assuming the EU28 electricity mix to a share of 90% in the case of stable transformation and 87% in the case of RNP delivery assuming a renewable electricity mix. Compared to the GHG emissions and primary energy demand of the molecular breeding technologies, the environmental impacts of the cultivation of Chicory variants and the processing to inulin (Hingsamer et al., 2022) are higher as these are reflecting an industrial value chain and a yearly production.

### 3.7 Economic impacts of the molecular breeding approaches

Additionally to the environmental assessment, direct and indirect economic impacts were calculated for the two breeding methods, RNP delivery (Method 1) and stable transformation (Method 3). In general, total costs for one experiment with three cycles were similar for both methods (16.7 thousand EUR per experiment for the RNP delivery and 18.4 thousand EUR per experiment for the stable transformation), with most of them concerning direct value-added (depreciation of capital and wages) while costs for material inputs with 20% for stable transformation and 32% for RNP delivery are small. In both methods around a third of total costs denote wages for scientists and other laboratory employees. In terms of material costs, electrical machinery, rubber, plastics and chemicals are the main inputs and cost categories (details are reported in **Supplementary Table 3**). Due to the similar height of costs, the total economic impacts do not differ much in height (**Table 5**) but in distribution across industries. In total stable transformation yields 41 thousand EUR of production of goods and services as well as 28 thousand EUR of direct and indirect value-added, while RNP delivery generates 43 thousand EUR of production of goods and services and 25 thousand EUR of direct and indirect value-added. Regarding employment effects, we find that the analyzed methods create around one job (number of persons) in the whole economy. In order to study in detail the nature of the impacts, final demand multipliers were also calculated (**Table 5**). Output multiplier with 4.1 and 4.2 respectively is similar in both methods, while the generated value-added is slightly higher in the stable transformation method. In particular, for 1 Euro in the development of the stable transformation 2.8 Euro value-added are generated, while the RNP delivery yields 2.4 Euro of value-added. This means, due to its higher labor intensity and slightly higher costs for one experiment the stable transformation has a higher value-added than RNP delivery in the application of the methods. Both methods show a high intensity of research and development intense activities. Due to their high labor intensity, higher employment and value-added multiplier effects compared to industrial or manufacturing processes. For instance, the job and value-added multiplier of the development of genome-editing methods are clearly higher than the respective multiplier of the industrial value chain processes based on the Chicory variants (Hingsamer et al., 2022). However, no conclusions can be drawn from the differences in value-added-multipliers about the efficiency of the methods, since the evaluation is aimed at the costs of experiments carried out.

**Table 5:** Economic effects of the two genome editing methods.

	Stable transformation	RNP delivery
Total impact on		
***Production of Good & Services (EUR)***	40,900	43,300
***Value added (EUR)***	27,600	25,100
***Jobs (number of persons)***	1.14	1.08
*Final demand multiplier:*		
***Output multiplier***	4.1	4.2
***Value added multiplier***	2.8	2.4
***Job multiplier***	0.072	0.067

Production output, value-added and job generated of the global economy in order to satisfy the final demand of all goods and services generated in the development of both genome-editing methods are reported.

## 4 Discussion

Three different CRISPR/Cas9 delivery methods were evaluated for their ability to introduce mutations into the Chicory genome: the use of a common guide RNA ensured that a comparison of all the methods could be made by looking at the efficiencies of the on-target and off-target mutations. The target chosen for the experiments was the small gene family of GAS genes in root Chicory, which is composed by four members (CiGAS-S1, -S2, -S3 and -L). Two of these genes matched perfectly with the designed guide RNA (CiGAS-S1 and -S2) while the other two can be considered as off-targets as they have each one, though different mismatch. Among the list of off-targets (**Supplementary Table 1**) they were the only ones with a single mismatch. With this particular experimental design it was possible not only to compare the different methods, but also the test the specificity in knocking out specific members of a gene family where the shared sequence homology is high (Bogdanović et al., 2019). Overall, all the methods were successful in the creation of many loss-of-function mutant lines in CiGAS-S1 and -S2, with no detectable off-target mutations in regions with two or more mismatches with the guide RNA. The single mismatch in CiGAS-S3 and -L did lead to editing although surprisingly, the mutation rate was high in -S3 (comparable to S1/S2) where the mismatch is close to the PAM, while it was low in -L where it stands in the middle of the sequence. These findings were consistent in all the three methods, contradict the assumption that mismatches in the seed region of the guide RNA have a higher impact on the editing efficiency (Modrzejewski et al., 2020). The first striking difference observed was between the transient (method 1 and 2) and stable (method 3) delivery: in the first case the genotypes were always well classifiable as mono- or bi-allelic (and in this case hetero or homozygous) (**Tables 1**, **2**), while in the second case the mutation profile was always chimeric to some extent (**Table 3**). Chimerism is deduced by the presence of wild type reads in the sequencing (indicating the presence of either untransformed cells, or cells in which Cas9 did not achieve any editing) or by the presence of more than two editing profiles in a locus. The lack of uniformity in the genotype of the plant may be a problem in case of vegetative propagation and can be fixed in principle only by other cycles of *in vitro* regeneration, or by reproduction through the germline (in Chicory complicated due to its self-incompatibility): both methods imply extra time, work, and costs. Another drawback of the stable transformation is the continuous expression of Cas9, which creates the possibility for new editing events during the lifetime of the plant, both in the wild type on-targets (therefore with a probable increase of chimerism) and in the off-targets. In fact, this was indeed detected in three independent lines that were sequenced 2 years apart (**Figure 3**). This timeframe was quite long and not likely to occur in a breeding programme that involves crossings, but it is representative of what may happen in the plant during the months necessary for the vegetative growth before the flowering period in the case a cross is needed to segregate away the T-DNA and erase chimerism. Hence, in chicory the methods based on transient delivery to protoplasts seemed more convenient as exposure to the editing complex can be controlled and because, although less simple to perform compared to the transformation with Agrobacterium, they lacked chimerism, did not have prolonged off-target activity and generally gave a higher number of regenerants, which are already a new variety that do not need further crossings to be used in the field. Indeed, both protoplast-based methods produced high numbers of plants with null alleles, but the transient plasmid approach (Method 2) led to a high percentage (a third) of plants with integrated plasmid DNA copies. It is relevant to note that this phenomenon might have been underestimated, since it is based on the detection by PCR of the Cas9 sequence, but not of other sequences in the plasmid. Considering that the design of primer pairs covering the whole plasmid is quite inconvenient, and assuming the possibility of plasmid rearrangements, the only way to prove absence of foreign DNA integration under Method 2 would be whole genome sequencing of the plants obtained after regeneration. Therefore, the data about plasmid DNA integration with this method should be taken as a lower estimate. Method 1 did not have this issue, because it was a DNA-free approach, where different guide RNAs could be easily produced or synthetized *in vitro*, making it very flexible for potentially tackling many genes. This last method may also be advantageous in the future: since no foreign DNA is introduced into the plant, mutants produced in this way might be subjected to a faster commercialization due to a simpler regulatory framework in some countries (Entine et al., 2021). Indeed, a crucial aspect to consider when choosing a method to create new varieties is the regulatory framework in the country in which the product is planned to be released. In Europe the above-mentioned techniques currently lead to plants considered as Genetically Modified Organisms (GMOs), no matter if foreign DNA is integrated into the genome or not. However, in other countries (Dima and Inzé, 2021) Method 1 could never result in a GMO, a status that plants obtain from Method 2 would need to be checked for plasmid integration and method 3 could achieve only by out-crossing the transgenes and proving thus the absence of exogenous DNA. From the environmental and economic point of view, the two methods examined show similar impacts but with difference distribution across sectors and therefore income distribution. The environmental impacts of molecular breeding are highly dependent on the electricity demand. Therefore, the use of electricity from renewable energy sources is a key factor for the contribution to decarbonization. Although total impacts on value-added and production of goods and services are modest, higher employment and value-added multiplier effects are created compared to common industrial or manufacturing processes. This traces back mainly due to the high labor intensity of R&D activities. Based on these considerations, from the comparison it can be concluded that the DNA-free approach from protoplasts is to be preferred, with the Agrobacterium-based method to be used only when other transformation methods cannot be implemented and/or plant regeneration from protoplasts cannot be achieved.

## Data availability statement

The datasets presented in this study can be found in online repositories. The names of the repository/repositories and accession number(s) can be found below: https://www.ncbi.nlm.nih.gov/genbank/, JAPIVL000000000.

## Author contributions

DB and KC conceived the research. KC, TS, PB, SM and MM designed the experiments. US, KU, PB, RS, MB, ST, KC, ES, RN and JH performed the experiments, collected the samples and analyzed data. MH, VK and MK performed the economic and environmental impact analysis. ES and RN sequenced and assembled the CHIC 2.0 genome. The manuscript was written by US, KU, MH, VK and revised by US, KU, PB, MB, KC, ES, RN, MH, VK, MK, DB, SM and MM. All authors contributed to the article and approved the submitted version.
