# Supplementary material for: A comparison of three different delivery methods for achieving CRISPR/Cas9 mediated genome editing in Cichorium intybus L
Source: Front Plant Sci. 2023 Apr 12;14:1111110. doi: 10.3389/fpls.2023.1111110 (PMC10131283; doi:10.3389/fpls.2023.1111110)
Supplement: Supplementary file 1 [file DataSheet_1.pdf]

Supplemetary Table 1. Putative off-targets found for the sgRNA targeting the four *Ci*GAS genes in chicory. The start and end point within the scaffold or contig, on which each off-target has been identified on is given. The annotation of possible genes by gene prediction algorithm using RNA-Seq Data from chicory and annotated genomes of related species is shown together with homologues genes. The PAM is marked in italic and mismatches between off-target side and sgRNA are shown in bold and underlined. Bold and italic off-target names are of special interest due to their annotation or low number of mismatches.

		sequence		mm	superscaffold/ contig	Start/End	position	GENE ID of related genes
WT	3' -	*CCT*TACATAAGGGATAGAGTACC	- 5'		-	-	-	
OT1	5' -	*CC****C***TACATAAGG**A**AT**C**GGGT**G**CC	- 3'	4	100108<br>100190	3013006-3013028<br>1090289-1090311	intergenic	
OT2	3' -	*CCT*TA**T**ATAA**A**GGATAGAGTACC	- 5'	4	100085	5062124-5062146	intergenic	
OT3	5' -	*CCA*TACATAA**T**GGATA**T**A**C**TAC**A**	- 3'	2	100235<br>100048	937474-937496<br>3609483-3609505	Exon	Lettuce_LSAT_1_V5_GN_7_8140.1.V5_R4<br>(F-box associated domain (FBA_3))
OT4	3' -	*CCA*TACATAAGG**A**AT**T**GA**AG**ACC	- 5'	4	100018	3131294-3131316	intergenic	
OT5	3' -	*CCC***A**ACATAA**A**GGATAGA**C**TACC	- 5'	4	100010	15270129-15270151	intergenic	
OT6	5' -	*CCC*TA**A**ATAAGG**A**AT**C**G**G**GTACC	- 3'	4	100108<br>100190	3162820-3162842<br>940859-940881	intergenic	
OT7	5' -	*CCC*T**C**CA**A**AA**A**G**A**ATAGAGTACC	- 3'	3	100108	5403976-5403998	intergenic	
OT8	3' -	*TCC*TACAT**T**AGGG**T**TAGAGTACC	- 5'	2	100031	1513597-1513619	intergenic	
OT9	5' -	*CCG***AG**CATAA**T**GGATAGAGT**T**CC	- 3'	4	100017	1067652-1067674	Exon	Sunflower_HANXRQCHR01G0011831.R1.2.494_R2<br>Lettuce_LSAT_1_V5_GN_5_96921.1.V5_R0
OT10	3' -	*CCC***A**A**G**ATAAGG**A**A**G**AGAGTACC	- 5'	4	100043	9692825-9692847	intergenic	
OT11	5' -	*CCA*T**C**CATAA**A**GGA**G**AGAGTACT	- 3'	2	Contig 20411	3524-3546	Exon	Lettuce_LSAT_1_V5_GN_8_93880.1.V5_R0
OT12	5' -	*CCA***C**A**G**ATA**GA**GGATAGAGTACC	- 3'	4	100113	177719-177741	intergenic	
OT13	3' -	*TCA*TACATAAGGGAT**T**GA**A**T**G**C**A**	- 5'	4	17716	16732712-16732734	Intron	Sunflower_HanXRQChr09g0269101.r1.2
OT14	3' -	*CCT*T**T**CATAAGG**ACA**AGAGTACC	- 5'	4	100008	18718286-18718308	intergenic	
OT15	3' -	*CCT*TA**T**ATAAGGG**T**TAGA**T**TA**G**C	- 5'	4	100240	742431-742453	intergenic	
OT16	3' -	*CCT*TACATAAGGG**T**T**TC**AGTA**G**C	- 5'	4	100066	7238486-7238508	intergenic	
OT17	3' -	*CCC*TACATAAG**A**GATA**A**A**C**TA**A**C	- 5'	4	100120	20515-20537	intergenic	
OT18	3' -	*CCC*TA**G**ATAA**AA**G**C**TAGAGTACC	- 5'	4	100008	669561-669583	intergenic	

Supplementary Table 2. List of primers used to amplify on- and off-target regions in chicory.

Name of primer pair	Sequence	Amplicon length	Description
S1-EX4-F/ S1-EX4-R	GGATTACTCATTTGTGAGCAAA/ TATGTCTTAGCATCATCGCTT	394 bp	*Ci*GAS-S1 Exon 4
S2-EX4-F/ S2-EX4-R	GGATTACTCATTTGTGAGCAAA/ CTTCTTAAGGATCATCGCTTAA	391bp	*Ci*GAS-S2 Exon 4
S1-KG-F/ S1-KG-R	TGTAAACCGTTGGGCTCGTC/ ACGCATGTGCTATAAACTC	1108 bp	Outer PCR *Ci*GAS-S1
S1/2-E4-Fw/ S1-E4-R	TTGAAGCGAAGTGTAAGGAT/ AGTCGAATCTCTTCAATGGT	254 bp	Inner PCR *Ci*GAS-S1 and S2
S2-KG-F/ S2-KG-R	CTATTAATCGGCTTTGGTCAAC/ GTTTGAGCGAGTCCATATGG	1704 bp	Outer PCR *Ci*GAS-S2
S3-KG-F/ S3-E4-R-New-2	GTTATTTCAGTTCCCCAAGTGT/ CATATGTGTCGTCTAGCAC	272 bp	PCR CiGAS-S3
L1b-E4-FW/ L1b-E4-RV	CGTGTCACAGTAAGTTTCATTTG/ CAGTGTTTTTGCGTATATATTAC	330 bp	PCR CiGAS-L
OT1_KU_FW/ OT1_KU_REV	AAGAAGACGGAGACCCAGAC/ TCAAAATGGTTGGCTATCCTCC	220 bp	PCR OT1
OT2_KU_FW/ OT2_KU_REV	GTGTTCTCCGCCGGTATAAATAA/ CCACCACCAGAAACAATGTAGTT	241 bp	PCR OT2
OT3_KU_FW/ OT3_KU_REV	CGGAGAGAGAAGGGTTCCAA/ TTGGTATTCTACGACGAGGC	191 bp	PCR OT3
OT4_KU_FW/ OT4_KU_REV	GGGATTCACTACTGTATCTAAGG/ ACATCGGTAACAAATTCGCATT	182 bp	PCR OT4
OT5_KU_FW/ OT5_KU_REV	TCGACACCTTATTTACTCAATCC/ CAGTTCTGGTGATGGCTTGT	228 bp	PCR OT5
OT6_KU_FW/ OT6_KU_REV	TATACAAGAAGACGGCGACC/ TGGAAAGATCAAAATGGTTGGT	226 bp	PCR OT6
OT7_KU_FW/ OT7_KU_REV	GCTGCTTTACATGTCATTCGATA/ ACAAACTCACTGCATGGATCT	250 bp	PCR OT7
OT8_KU_FW/ OT8_KU_REV	TATTCGAGCATCTTGGGACG/ TGATGGAAGGTGTGGACAAA	240 bp	PCR OT8
OT9_KU_FW/ OT9_KU_REV	AGCTTCGATTGTCACCATTCT/ ACAATTGTTAGACCTCACCA	201 bp	PCR OT9
OT10_KU_FW/ OT10_KU_REV	AGTGTCTTCAAGAAAAGGAAACA/ ACTTCCCAGATGTTGAAAGG	242 bp	PCR OT10
OT11_KU_FW/ OT11_KU_REV	CGTTTGGTGAGACTCCTTCG/ CGCTAAACAGTTCCTCGAGG	202 bp	PCR OT11
OT12_KU_FW/ OT12_KU_REV	ACCAAATCAAAACCAAACATACA/ ATCGAAATGACTTTGCGTGC	229 bp	PCR OT12
OT13_KU_FW/ OT13_KU_REV	AATGAAGTGAGTGGTTACCA/ GAATGTCAAGTTGCCATTTA	219 bp	PCR OT13
OT14_KU_FW/ OT14_KU_REV	TGCCCCAATCTCTGATACCA/ CACCTTTGCGTTGCTACTTG	240 bp	PCR OT14
OT15_KU_FW/ OT15_KU_REV	CGGTTAAGCGGTCGATCC/ ACCCAATCAAACCGAAACCT	226 bp	PCR OT15
OT16_KU_FW/ OT16_KU_REV	CAGGCATCCACATCTATAAGCA/ TGACCTTGGCTTGGATCCA	136 bp	PCR OT16
OT17_KU_FW/ OT17_KU_REV	GGGAAGGAAATGAAAAGAACTTT/ TTTGGGTTTTGCTAAATTGC	241 bp	PCR OT17
OT18_KU_FW/ OT18_KU_REV	GCACTTTGGAAGCTTGTCGA/ CTTCTTTCTTTCCTCCCCGC	206 bp	PCR OT18

Supplementary Table 3. Specification of cost vector for respective molecular breeding method.

	Stable transformation		*RNP delivery*	
	Costs in €	*Cost share in %*	*Costs in €*	*Cost share in %*
Crops	494	7%	152	1%
Chemicals	572	9%	1,289	8%
Rubber and plastic products	765	11%	769	5%
Machinery and equipment	27	0%	22	0%
Office machinery and computers	9	0%	9	0%
Electrical machinery and apparatus	1,293	19%	1,153	7%
Electricity	60	1%	702	4%
Other land transportation services	255	4%	255	2%
Research and development services	240	4%	1,032	6%
Sum of intermediate inputs	**3,715**	20%	**5,382**	32%
Value-added	**14,726**	80%	**11,328**	68%
Total costs	**18,442**		**16,710**	

Supplementary Table 4. CHIC 2.0 genome sequencing and assembly data.

**Pacbio Sequence data**	data set 1	data set 2	BUSCO	complete single BUSCO's	complete duplicated BUSCO's
Mean Read Length	13.184	11.381			
N50 Read Length	23.266	17.832			
Number of reads	4.242.764	2.673.205			
Total bases	55.936.200.299	30.424.184.294			
**Assembly (Flye 2.5)**			94,7%	1.418	785
Number of sequences	17.113				
Total sequence length	1.548.700.630				
Average sequence length	90.498				
Maximum sequence length	8.001.753				
N50 sequence index	1.081				
N50 sequence length	384.198				

**Polished assembly**					
Number of sequences	17.113				
Total sequence length	1.551.705.774				
Average sequence length	90.674				
Maximum sequence length	8.737.564				
N50 sequence index	1.083				
N50 sequence length	384.670				

**Purged assembly**			94,0%	1.823	364
	Haploid assembly	Assigned haplotigs			
Number of sequences	8.773	8.340			
Total sequence length	1.249.143.101	302.562.673			
AV sequence length	142.385	36.278			
Max sequence length	8.737.564	1.516.748			
N50 sequence length	468.930	153.531			
N50 sequence index	741	555			

**Bionano Genomics data**	
Input molecule stats (filtered)	
Total number of molecules	639.449
Total length (Mbp)	198.008
Average length (kbp)	309,7
Molecule N50 (kbp)	313,6
Label density (/100kb)	11,7

Genome map De novo assembly

Count = 475	475
Min length (Mbp)	0,24
Median length (Mbp)	3,12
Mean length (Mbp)	5,29
N50 length (Mbp)	9,64
Max length (Mbp)	37,42
Total length (Mbp)	2.514,86

Molecules aligned to the assembly:

Total number of molecules aligned	438.284
Fraction of molecules aligned	0,56
Effective coverage of assembly (X)	51,8

Hybrid scaffold FASTA statistics (CHIC v2.0):

Count	332
Min length (Mbp)	0,10
Median length (Mbp)	3,14
Mean length (Mbp)	5,30
N50 length (Mbp)	9,81
Max length (Mbp)	53,97
Total length (Mbp)	1.761,0

Hybrid scaffold FASTA plus not scaffolded NGS FASTA statistics:

Count	6.649
Min length (Mbp)	0
Median length (Mbp)	0,01
Mean length (Mbp)	0,28

N50 length (Mbp)	8,88
Max length (Mbp)	53,97
Total length (Mbp)	1.872,71

Supplementary Figure 1. A50 plot of Cichorium intybus clone 37 purged assembly (red) and hybrid scaffolds of assembly CHIC v2.0 (blue). Graph shown is based on cumulative assembly graph of fragments > 2500 bp.

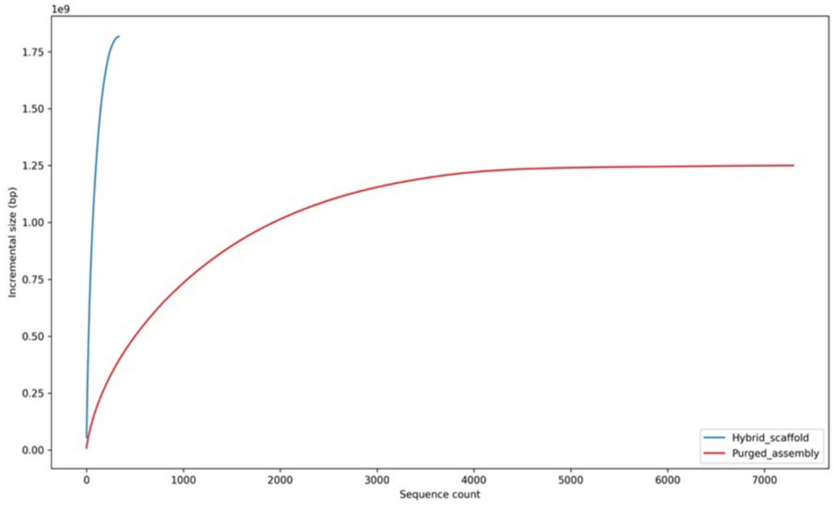

Supplementary Figure 2. Mummer plot of Cichorium intybus clone 37 purged assembly (y-axis) versus public Cichorium intybus assembly GCA_023525715.1 (x-axis).

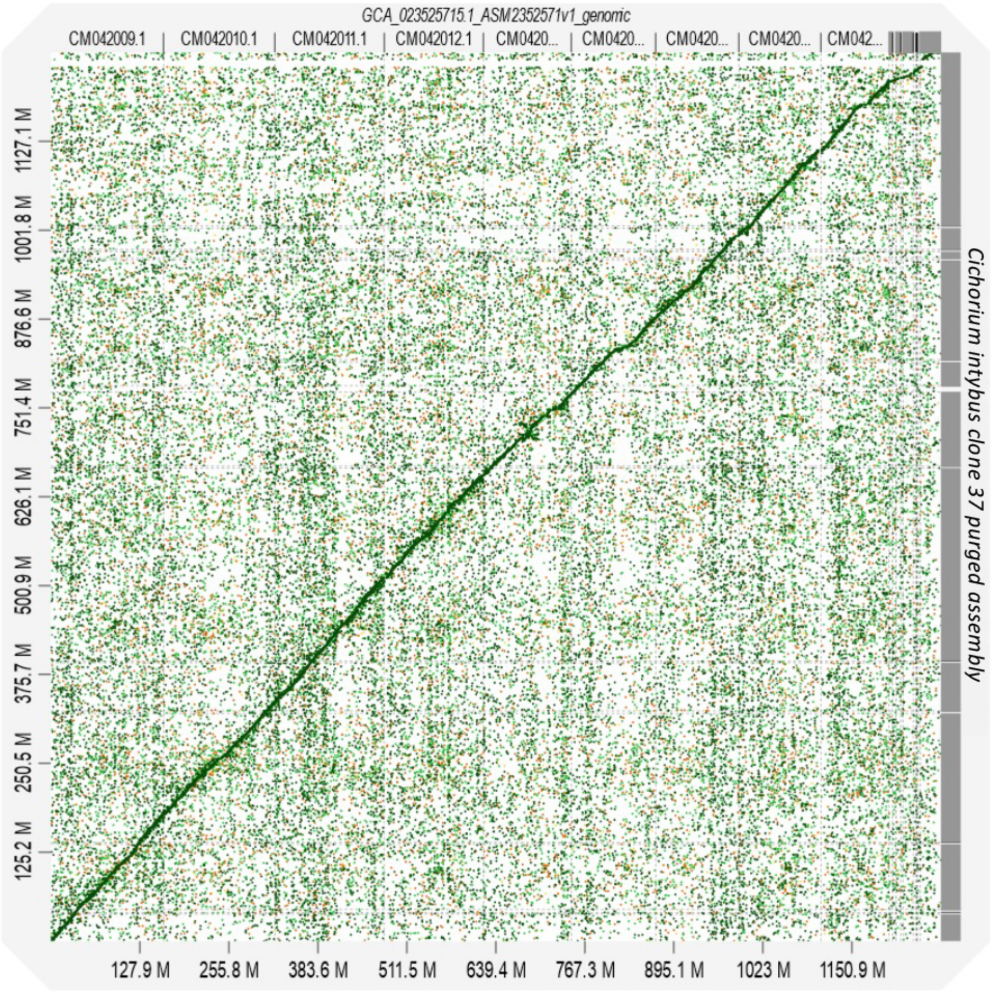

Supplementary Figure 3. CG-Backbone1 plasmid map and sequence used for Method 3 (Stable transformation).

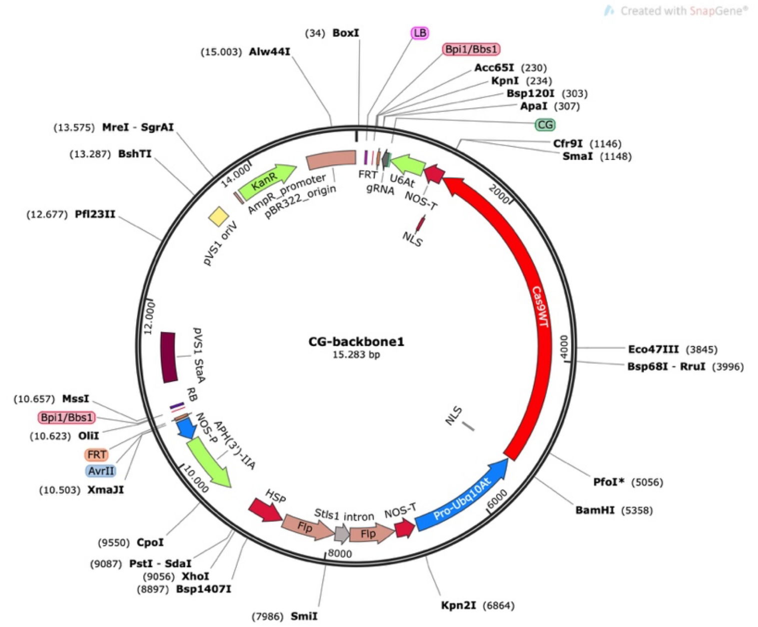

>CG-Backbone1
tacggttcctgctcggatctgttggaccggacagtagtcatggttgatgggctgcctgtatcgagtggtgattttgtgcc
gagctgccggtcggggagctgttggctggctggtggcaggatatattgtggtgtaaacaaattgacgcttagacaactta
ataacacattgcggacgtttttaatgtactggggttgaacactctgtgccttgtcttccagtggtatggggtaccggggc
gtaggtaatgtccacctcgatatgtgcggaagttcctattctctagaaagtataggaacttcgggcccaagatctggccc
ttaaggcctgtcgacaaaaaaagcaccgactcggtgccactttttcaagttgataacggactagccttattttaacttgc
tatttctagctctaaaacTACATAAGGGATAGAGTACCaatcactacttcgactctagctgtatataaactcagcttcgt
tttcttatctaagcgatgtgggacttttgaagattgttttcaacttaaatgggcctatataagaaatactattgttcttt
cccatataaatgggcctgcttctcttctttcagattcccaggggccttttgaagattatcttcatatcttaagaatgaag
atgttttattcaatcaaattcttgaaggttcgatgcctaatcattctaatcctgggacaaactatgaaacaagatacaaa
aactccgaatggaaagttaaaaagaagaaaacgaaagctacggttcaagaaaatgtaagctgataaacaaaaaaaaactg
tatgaacgaagaagaagaaaaaaagctaagaagaaatgatgtattgtgcggaaggcaagtcgagtttccgttgttcaacg
aaggaattcgagcgatctagtaacatagatgacaccgcgcgcgataatttatcctagtttgcgcgctatattttgttttc
tatcgcgtattaaatgtataattgcgggactctaatcataaaaacccatctcataaataacgtcatgcattacatgttaa
ttattacatgcttaacgtaattcaacagaaattatatgataatcatcgcaagaccggcaacaggattcaatcttaagaaa
ctttattgccaaatgtttgaacgatcccgggttacttcttcttcttggcctggccagccttcttggtggcggcggggcgc
ttgtctccgccgagttgggacaggtcgatcctggtttcgtacaacccagtaatgctctggtggataagggtagcatcgag
cacttccttggtggaggtgtaacgcttgcggtcgatggtagtgtcgaagtacttgaaggcggctggggcacccaggttgg
tgagggtgaacaagtggataatgttttcagcctgctctctgataggcttgtccctgtgcttgttgtaggcagaaaggact
ttatcgagattggcgtcagccaggataacgcgcttggagaactcgctgatctgctcaatgatctcgtcaaggtagtgctt
atgctgctccacaaacaactgcttctgttcgttgtcctcggggctgcccttgagcttctcatagtgggaggcgaggtaca
ggaagttgacgtacttgcttgggagggccaactcgtttccctttttgcagctcgccagcggaggcgagcatacgcttcctt
ccgttctcaagctcgaacagagagtacttggggagcttgatgatcaagtccttcttcacttccttgtagcccttggcctc
aaggaaatcaatagggttcttctcgaaggagctgcgctccatgatggtgatcccgagcagctccttgacggacttgagct
tcttgctcttgcccttctcaactttagccacgacaagcacggagtaggcgacggttgggctgtcgaatccaccgtacttc
ttggggtcccagtccttcttacgggcaatcaacttatcggaattgcgctttgggaggatagactccttggagaagcctcc
agtctgaacctcggttttcttcacgatgttgacttggggcatgctcagcacttttctgacggtggcgaagtccctgccct
tgtcccacacgatctcgccggtttctccgttggtttcaatgagagggcgcttacggatctccccgttagccaaagtgatc
tcggtcttaaagaagttcatgatgttggagtagaagaagtacttggcggtggccttgccaatctcctgctcgctcttagc
gatcatcttgcgaacgtcgtagaccttgtagtcaccatacacgaactcggactccagcttggggtacttcttgatcaagg
cggtgccgaccacggcattaaggtaagcatcgtgggcgtggtggtagttgttgatctccctaaccttgtagaattggaag
tcctttctgaagtcagacacgagcttgctcttgagggtaatcactttgacctcgcggatcagcttgtcgttctcatcgta
cttagtgttcatacgggagtcgaggatctgggccacgtgcttggtaatctggcgggtttcaacaagttgcctcttgatga
atccagccttgtccagttcgctgaggcctccacgctcggccttggtcaagttgtcgaacttgcgctgggtgatgagcttg

gcgttaagcagctgcctccagtaattcttcatcttcttgaccacctcctcggatgggacgttgtcggacttgccgcggtt
cttatcgcttctagtcaacaccttgttgtcgatagagtcgtccttgagaaaggactgagggacaatgtgatccacgtcgt
agtcgctgaggcggttgatgtccagctcttggtcaacgtacatatcgcgcccgttctgaaggtagtacaaatacagcttc
tcattctggagttgggtgttttccacggggtgctccttgaggatctgggaaccaagctccttgatgccctcctcaatcct
cttcatgcgctcacgggagttcttctgtcccttctgggtggtctggttttccctggccatctcgatgacgatgttctctg
gcttatgtctgcccatcaccttgaccaactcgtcaaccaccttgacggtctggaggattcccttcttaatagcggggctc
ccggccaggttggcgatgtgctcgtggaggctgtcgccctggccggacacttgagccttttggatgtcctccttgaaggt
aagagaatcgtcgtggatcagctgcatgaagttgcgattggcgaatccgtcggacttcaagaagtcgagaatagtcttgc
cgctctgcttgtcgcggataccgttgatgagcttacgggacagcctgccccatccggtgtagcgcctacgcttaagttgc
ttcatgactttatcgtcgaacaagtgggcgtaggtcttgaggcgttcctcaatcatctctctgtcctcgaacagggtgag
ggtaagaacgatgtcctccaagatatcttcgttctcctcgttgtcgaggaagtccttgtccttgataatcttcaggaggt
cgtggtaagtgcccaaggaagcgttgaagcgatcctccactccgctgatctcgacagagtcaaagcactcgatcttcttg
aagtagtcctccttaagctgcttcacggtgactttcctgttggtcttgaacaggaggtcaacgatggccttcttctgctc
gccgctgaggaaggcaggcttacgcataccctcggtcacgtacttcactttggtcaactcattgtagacagtgaagtact
cgtacagaagggagtgctttgggaggactttctcgttgggcaggttcttatcgaagttggtcatgcgctcaatgaaggac
tgagcgctggccccttgtcaaccacctcctcgaagttccatggggtgatggtttcctcggacttcctggtcatccaggc
gaagcggctgttgccacgggccaagggtccgacgtagtaagggattctaaaagtgaggatcttttcaatcttctcgcgat
tgtccttaaggaatggatagaagtcctcttgcctgcggaggatagcgtgcagctcgccgagatggatctggtggggaata
gatccgttgtcgaaggtacgctgcttcctcaaaagatcctcgcggttgagcttcacgagcagctcttcggtgccgtccat
cttctccaagatgggcttgatgaacttgtagaactcctcttgggaggccccgccgtcgatgtaaccggcgtatccgttct
tgctctggtcgaagaaaatctccttgtacttctcgggcagctgctgtctgacaagagccttgaggagggtcaaatcttgg
tggtgttcgtcgtaacgcttgatcatggaggcgctcagtggggccttggtgatctcagtgttcacgcgaaggatgtcgga
gagcagaatagcgtcggagaggttcttggcggccaagaaaaggtcagcgtactgatcgccgatctgggcgagcaggttgt
ccaagtcgtcatcgtaggtgtccttgctgagttgcagcttggcgtcctcggcgaggtcaaaattagacttgaagttaggg
gtaaggcccaaggagagagcgatcaggtttccgaagaggccgttcttcttctctcctgggagctgggcgatcaagttctc
aagcctgcggctcttggacagacgggcgctgagaatagccttggcgtcaaccccggaggcgttgataggattctcctcga
aaagctggttgtaggtctggaccagttggatgaacaacttatcgacgtcagagttgtcggggttgaggtcgccctcaatc
aggaagtgaccgcggaacttgatcatgtgagcgagggcaaggtagatgagcctcaaatcggccttgtcggtggagtccac
cagcttctttctgaggtggtagatagttggatacttctcgtggtaagccacctcgtcaacaatgtttccgaagatgggat
ggcgctcgtgcttcttgtcctcctcgacaaggaagctctcctccaaacggtgaaagaaggaatcgtccactttggccatc
tcgttgctgaagatctcctggaggtagcagatcctattcttgcgacgggtgtagcgccttctggcggtgcgcttcagacg
ggtggcctcagcggtttcgccggagtcgaagagaagggcgccaatcaagttcttcttgatgctgtgcctgtcagtgtttc
cgaggactttgaacttcttagaaggcactttgtactcatcggtgataacggcccagccgacggagttggtcccgatgtcc
agaccaatgctgtacttcttgtctggagctccgtggattcccactttgcgcttcttcttgggggccatagttatggagga
tccactagtacctgttaatcagaaaaactcagattaatcgacaaattcgatcgcacaaactagaaactaacaccagatct
agatagaaatcacaaatcgaagagtaattattcgacaaaactcaaattatttgaacaaatcggatgatatctatgaaacc
ctaatcgagaattaagatgatatctaacgatcaaacccagaaaatcgtcttcgatctaagattaacagaatctaaaccaa
agaacatatacgaaattgggatcgaacgaaaacaaaatcgaagattttgagagaataaggaacacagaaatttaccttga
tcacggtagagagaattgagagaaagtttttaagattttgagaaattgaaatctgaattgtgaagaagaagagctctttg
ggtattgttttatagaagaagaagaagaaaagacgaggacgactaggtcacgagaaagctaaggcggtgaagcaatagct
aataataaaatgacacgtgtattgagcgttgtttacacgcaaagttgtttttggctaattgccttatttttaggttgagg
aaaagtatttgtgctttgagttgataaacacgactcgtgtgtgccggctgcaaccactttgacgccgtttattactgact
cgtcgacaaccacaatttctaacggtcgtcataagatccagccgttgagatttaacgatcgttacgatttatattttttt
agcattatcgttttatttttaaatatacggtggagctgaaaattggcaataattgaaccgtgggtcccactgcattgaa
gcgtatttcgtattttctagaattcttcgtgctttatttcttttcctttttgttttttttgccatttatctaatgcaag
tgggcttataaaatcagtgaatttcttggaaaagtaacttctttatcgtataacatattgtgaaattatccatttctttt
aatttttagtgttattggatatttttgtatgattattgatttgcataggataatgacttttgtatcaagttggtgaaca
agtctcgttaaaaaaggcaagtggtttggtgactcgatttattcttgttatttaattcatatatcaatggatcttatttg
gggcctggtccatatttaacactcgtgttcagtccaatgaccaataatatttttttcattaataacaatgtaacaagaatg
atacacaaaacattctttgaataagttcgctatgaagaagggaacttatccggtcctagatcatcagttcatacaaacct
ccatagagttcaacatcttaaacaagaatatcctgatctgaagaatgtggaggctttagtcccttggatacttgggaggc
tgtggaagaacagaaacgagctggtgcttaaagggagggaatttggaaccaatgaggtattagtaaggacacaagaagat
gcagatgagtggattagaaggaaagaggctcagaatgtaaggaaagcggcggccgccatggcctccggacgagcgatcta
gtaacatagatgacaccgcgcgcgataatttatcctagtttgcgcgctatattttgttttctatcgcgtattaaatgtat
aattgcgggactctaatcataaaaacccatctcataaataacgtcatgcattacatgttaattattacatgcttaacgta
attcaacagaaattatatgataatcatcgcaagaccggcaacaggattcaatcttaagaaactttattgccaaatgtttg
aacgatcgcgcgcctcatatgcgtctatttatgtaggatgaaaggtagtctagtacctcctgtgatattatcccattcca

tgcggggtatcgtatgcttccttcagcactaccctttagctgttctatatgctgccactcctcaattggattagtctcat
ccttcaatgctatcatttcctttgatattggatcatatgcatagtaccgagaaactagtgcgaagtagtgatcaggtatt
gctgttatctgatgagtatacgttgtcctggccacggcagaagcacgcttatcgctccaatttcccacaacattagtcaa
ctccgttaggcccttcattgaaagaaatgaggtcatcaaatgtcttccaatgtgagattttgggccatttttatagcaa
agattgaataaggcgcatttttcttcaaagctttattgtacgatctgactaagttatcttttaataattggtattcctgt
ttattgcttgaagaattgccggtcctatttactcgttttaggactggttcagaattcctcaaaaattcatccaaatatac
aagtggatcgatcctacccccttgcgctaaagaagtatatgtgcctgcacatcaacaaattttggtcatatattagaaaag
ttataaattaaaatatacacacttataaactacagaaaagcaattgctatatactacattcttttattttgaaaaaaata
tttgaaatattatattactactaattaatgataattattatatatatatcaaaggtagaagcagaaacttacctgctaac
gcttgtctttgtctctgtcactaaacactggattattactcccagatacttattttggactaatttaaatgatttcggat
caacgttcttaatatcgctgaatcttccacaattgatgaaagtagctaggaagaggaattggtataaagtttttgttttt
gtaaatctcgaagtatactcaaacgaatttagtattttctcagtgatctcccagatgctttcaccctcacttagaagtgc
tttaagcatttttttactgtggctatttcccttatctgcttcttccgatgattcgaactgtaattgcaaactacttacaa
tatcagtgatatcagattgatgtttttgtccatagtaaggaataattgtaaattcccaagcaggaatcaatttctttaat
gaggcttccagaattgttgctttttgcgtcttgtatttaaactggagtgatttattgacaatatcgaaactcagcgaatt
gcttatgatagtattatagctcatgaatgtggctctcttgattgctgttccgttatgtgtaatcatccaacataaatagg
ttagttcagcagcacataatgctattttctcacctgaaggtctttcaaacctttccacaaactgacgaacaagcacctta
ggtggtgttttacataatatatcaaattgtggcatgcttagacgtcaccgcaggtaagcttgatacgatgccttcacttc
tttctgtgatgtgtcttcaaaggacacatggggtatttaaaatgatgtagaggagcaatatagtactttcgagaaagtcc
ttgttatgcatcatcatgccgtacttctgaaaatctcaaaactagaattttctgaaactttcagaaacattctggacttc
ttcgaaccgcaactagaagtttcgggattggaccatcttaaccagacattggttaggattggtctaccaggcccaaattg
cctttatggtgggctttgtacaaatgattttttttttttttattaatgagtctaataagtaaacatcgtatttaaagagt
gagataggatgagttgaacttaatttataaatttgtatttttaataatgttttacgcaaattatattttaaattctatct
cttaaaaggctgactctcgaGaagcttggcgatcgaattctcctgcaggcttcatcagcttgcatgccggtcgatctagt
aacatagatgacaccgcgcgcgataatttatcctagtttgcgcgctatattttgttttctatcgcgtattaaatgtataa
ttgcgggactctaatcaaaaaacccatctcataaataacgtcatgcattacatgttaattattacatgcttaacgtaatt
caacagaaattatatgataatcatcgcaagaccggcaacaggattcaatcttaagaaactttattgccaaatgtttgaac
gatctgcttgactctagctagagtccgaaccccagagtcccgctcagaagaactcgtcaagaaggcgatagaaggcgatg
cgctgcgaatcgggagcggcgataccgtaaagcacgaggaagcggtcagcccattcgccgccaagctcttcagcaatatc
acgggtagccaacgctatgtcctgatagcggtccgccacacccagccggccacagtcgatgaatccagaaaagcggccat
tttccaccatgatattcggcaagcaggcatcgccctgggtcacgacgagatcctcgccgtcgggcatccgcgccttgagc
ctggcgaacagttcggctggcgcgagcccctgatgctcttcgtccagatcatcctgatcgacaagaccggcttccatccg
agtacgtcctcgctcgatgcgatgtttcgcttggtggtcgaatgggcaggtagccggatcaagcgtatgcagccgccgca
ttgcatcagccatgatggatactttctcggcaggagcaaggtgagatgacaggagatcctgccccggcacttcgcccaat
agcagccagtcccttcccgcttcagtgacaacgtcgagcacagctgcgcaaggaacgcccgtcgtggccagccacgatag
ccgcgctgcctcgtcttggagttcattcagggcaccggacaggtcggtcttgacaaaaagaaccgggcgcccctgcgctg
acagccggaacacggcggcatcagagcagccgattgtctgttgtgcccagtcatagccgaatagcctctccacccaagcg
gccggagaacctgcgtgcaatccatcttgttcaatcatgcctcgatcgagttgagagtgaatatgagactctaattggat
accgaggggaatttatggaacgtcagtggagcatttttgacaagaaatatttgctagctgatagtgaccttaggcgactt
ttgaacgcgcaataatggtttctgacgtatgtgcttagctcattaaactccagaaacccgcggctgagtggctccttcaa
cgttgcggttctgtcagttccaaacgtaaaacggcttgtcccgcgtcatcggcgggggtcataacgtgactcccttaatt
ctcatgtatgataattcgcggtCctaggaagttcctattctctagaaagtataggaacttcaattcaagcttggagtacg
ttaattagttgtctgttttaagccccgtgctctccgccggggctttttttattaaaaaccactcaagtggaagacaaggga
cacgaagtgatccgtttaaactatcagtgtttgacaggatatattggcgggtaaacctaagagaaaagagcgtttattag
aataatcggatatttaaaagggcgtgaaaaggtttatccgttcgtccatttgtatgtgcatgccaaccacagggttcccc
tcgggagtcagccgtgcggctgcatgaaatcctggccggtttgtctgatgccaagctggcggcctggccggccagcttgg
ccgctgaagaaaccgagcgccgccgtctaaaaaggtgatgtgtatttgagtaaaacagcttgcgtcatgcggtcgctgcg
tatatgatgcgatgagtaaataaacaaatacgcaaggggaacgcatgaaggttatcgctgtacttaaccagaaaggcggg
tcaggcaagacgaccatcgcaacccatctagcccgcgccctgcaactcgccggggccgatgttctgttagtcgattccga
tccccagggcagtgcccgcgattgggcggccgtgcgggaagatcaaccgctaaccgttgtcggcatcgaccgcccgacga
ttgaccgcgacgtgaaggccatcggccggcgcgacttcgtagtgatcgacggagcgccccaggcggcggacttggctgtg
tccgcgatcaaggcagccgacttcgtgctgattccggtgcagccaagcccttacgacatatgggccaccgccgacctggt
ggagctggttaagcagcgcattgaggtcacggatggaaggctacaagcggcctttgtcgtgtcgcgggcgatcaaaggca
cgcgcatcggcggtgaggttgccgaggcgctggccgggtacgagctgcccattcttgagtcccgtatcacgcagcgcgtg
agctacccaggcactgccgccgccggcacaaccgttcttgaatcagaacccgagggcgacgctgcccgcgaggtccaggc
gctggccgctgaaattaaatcaaaactcatttgagttaatgaggtaaagagaaaatgagcaaaagcacaaacacgctaag
tgccggccgtccgagcgcacgcagcagcaaggctgcaacgttggccagcctggcagacacgccagccatgaagcgggtca

actttcagttgccggcggaggatcacaccaagctgaagatgtacgcggtacgccaaggcaagaccattaccgagctgcta
tctgaatacatcgcgcagctaccagagtaaatgagcaaatgaataaatgagtagatgaattttagcggctaaaggaggcg
gcatggaaaatcaagaacaaccaggcaccgacgccgtggaatgccccatgtgtggaggaacgggcggttggccaggcgta
agcggctgggttgtctgccggccctgcaatggcactggaacccccaagcccgaggaatcggcgtgacggtcgcaaaccat
ccggcccggtacaaatcggcgcggcgctgggtgatgacctggtggagaagttgaaggccgcgcaggccgcccagcggcaa
cgcatcgaggcagaagcacgccccggtgaatcgtggcaagcggccgctgatcgaatccgcaaagaatcccggcaaccgcc
ggcagccggtgcgccgtcgattaggaagccgcccaagggcgacgagcaaccagattttttcgttccgatgctctatgacg
tgggcacccgcgatagtcgcagcatcatggacgtggccgttttccgtctgtcgaagcgtgaccgacgagctggcgaggtg
atccgctacgagcttccagacgggcacgtagaggtttccgcagggccggccggcatggccagtgtgtgggattacgacct
ggtactgatggcggtttcccatctaaccgaatccatgaaccgataccgggaagggaagggagacaagcccggccgcgtgt
tccgtccacacgttgcggacgtactcaagttctgccggcgagccgatggcggaaagcagaaagacgacctggtagaaacc
tgcattcggttaaacaccacgcacgttgccatgcagcgtacgaagaaggccaagaacggccgcctggtgacggtatccga
gggtgaagccttgattagccgctacaagatcgtaaagagcgaaaccgggcggccggagtacatcgagatcgagctagctg
attggatgtaccgcgagatcacagaaggcaagaacccggacgtgctgacggttcaccccgattactttttgatcgatccc
ggcatcggccgttttctctaccgcctggcacgccgcgccgcaggcaaggcagaagccagatggttgttcaagacgatcta
cgaacgcagtggcagcgccggagagttcaagaagttctgtttcaccgtgcgcaagctgatcgggtcaaatgacctgccgg
agtacgatttgaaggaggaggcggggcaggctggcccgatcctagtcatgcgctaccgcaacctgatcgagggcgaagca
tccgccggttcctaatgtacggagcagatgctagggcaaattgccctagcaggggaaaaaggtcgaaaaagcttctttcc
tgtggatagcacgtacattgggaacccaaagccgtacattgggaaccggaacccgtacattgggaacccaaagccgtaca
ttgggaaccggtcacacatgtaagtgactgatataaaagagaaaaaaggcgatttttccgcctaaaactctttaaaactt
attaaaactcttaaaacccgcctggcctgtgcataactgtctggccagcgcacagccgaacagctgcaaaaagcgcctac
ccttcggtcgctgcgctccctacgccccgccgcttcgcgtcggcctatcgcggccgctggccgctcaaaaatggctggcc
tacggccaggcaatctaccagggcgcggacaagccgcgccgtcgccactcgaccgccggcgcccacatcaaggctccgag
tgcgcggaacccctatttgtttatttttctaaatacattcaaatatgtatccgctcatgagacaataaccctgataaatg
cttcaataatattgaaaaaggaagagtatggctaaaatgagaatatcaccggaattgaaaaaactgatcgaaaaataccg
ctgcgtaaaagatacggaaggaatgtctcctgctaaggtatataagctggtgggagaaaatgaaaacctatatttaaaaa
tgacggacagccggtataaagggaccacctatgatgtggaacgggaaaaggacatgatgctatggctggaaggaaagctg
cctgttccaaaggtcctgcactttgaacggcatgatggctggagcaatctgctcatgagtgaggccgatggcgtcctttg
ctcggaagagtatgaagatgaacaaagccctgaaaagattatcgagctgtatgcggagtgcatcaggctctttcactcca
tcgacatatcggattgtccctatacgaatagcttagacagccgcttagccgaattggattacttactgaataacgatctg
gccgatgtggattgcgaaaactgggaagaggacactccatttaaagatccgcgcgagctgtatgatttttttaaagacgga
aaagcccgaagaggaacttgtcttttcccacggcgacctgggagacagcaacatctttgtgaaagatggcaaagtaagtg
gctttattgatcttgggagaagcggcagggcggacaagtggtatgacattgccttctgcgtccggtcgctcagggaggat
atcggggaagaacagtatgtcgagctatttttgacttactggggatcaagcctgattgggagaaaataaaatattatat
tttactggatgaattgttttagctgtcagaccaagtttactcatatatactttagattgatttaaaacttcatttttaat
ttaaaaggatctaggtgaagatcctttttgataatctcatgaccaaaatcccttaacgtgagttttcgttccactgagcg
tcagaccccgtagaaaagatcaaaggatcttcttgagatcctttttttctgcgcgtaatctgctgcttgcaaacaaaaaa
accaccgctaccagcggtggtttgtttgccggatcaagagctaccaactctttttccgaaggtaactggcttcagcagag
cgcagataccaaatactgttcttctagtgtagccgtagttaggccaccacttcaagaactctgtagcaccgcctacatac
ctcgctctgctaatcctgttaccagtggctgctgccagtggcgataagtcgtgtcttaccgggttggactcaagacgata
gttaccggataaggcgcagcggtcgggctgaacggggggttcgtgcacacagcccagcttggagcgaacgacctacaccg
aactgagatacctacagcgtgagctatgagaaagcgccacgcttcccgaagggagaaaggcggacaggtatccggtaagc
ggcagggtcggaacaggagagcgcacgagggagcttccagggggaaacgcctggtatctttatagtcctgtcgggtttcg
ccacctctgacttgagcgtcgatttttgtgatgctcgtcaggggggcggagcctatggaaaaacgccagcaacgcggcct
ttt

Supplementary Figure 4. CG-Backbone2 plasmid map and sequence used for Method 2 and 3 (Transient and Stable transformation).

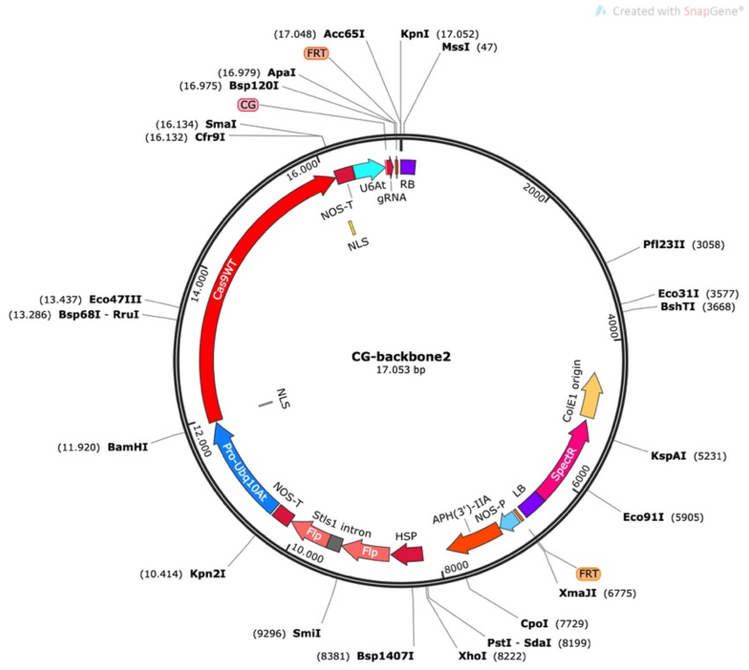

>CG-Backbone2

agcttagcttgagcttggatcagattgtcgtttcccgccttcagtttaaactatcagtgtttgacaggatatattggcgg
gtaaacctaagagaaaagagcgtttattagaataacggatatttaaaagggcgtgaaaaggtttatccgttcgtccattt
gtatgtgcatgccaaccacagggttcccctcgggatcaaagtactttgatccaacccctccgctgctatagtgcagtcgg
cttctgacgttcagtgcagccgtcttctgaaaacgacatgtcgcacaagtcctaagttacgcgacaggctgccgccctgc
ccttttcctggcgttttcttgtcgcgtgttttagtcgcataaagtagaatacttgcgactagaaccggagacattacgcc
atgaacaagagcgccgccgctggcctgctgggctatgcccgcgtcagcaccgacgaccaggacttgaccaaccaacgggc
cgaactgcacgcggccggctgcaccaagctgttttccgagaagatcaccggcaccaggcgcgaccgcccggagctggcca
ggatgcttgaccacctacgccctggcgacgttgtgacagtgaccaggctagaccgcctggcccgcagcacccgcgaccta
ctggacattgccgagcgcatccaggaggccggcgcgggcctgcgtagcctggcagagccgtgggccgacaccaccacgcc
ggccggccgcatggtgttgaccgtgttcgccggcattgccgagttcgagcgttccctaatcatcgaccgcacccggagcg
ggcgcgaggccgccaaggcccgaggcgtgaagtttggcccccgccctaccctcaccccggcacagatcgcgcacgcccgc
gagctgatcgaccaggaaggccgcaccgtgaaagaggcggctgcactgcttggcgtgcatcgctcgaccctgtaccgcgc
acttgagcgcagcgaggaagtgacgcccaccgaggccaggcggcgcggtgccttccgtgaggacgcattgaccgaggccg
acgccctggcggccgccgagaatgaacgccaagaggaacaagcatgaaaccgcaccaggacggccaggacgaaccgtttt
tcattaccgaagagatcgaggcggagatgatcgcggccgggtacgtgttcgagccgcccgcgcacgtctcaaccgtgcgg
ctgcatgaaatcctggccggtttgtctgatgccaagctggcggcctggccggccagcttggccgctgaagaaaccgagcg
ccgccgtctaaaaaggtgatgtgtatttgagtaaaacagcttgcgtcatgcggtcgctgcgtatatgatgcgatgagtaa
ataaacaaatacgcaaggggaacgcatgaaggttatcgctgtacttaaccagaaaggcgggtcaggcaagacgaccatcg
caacccatctagcccgcgccctgcaactcgccggggccgatgttctgttagtcgattccgatccccagggcagtgcccgc
gattgggcggccgtgcgggaagatcaaccgctaaccgttgtcggcatcgaccgcccgacgattgaccgcgacgtgaaggc
catcggccggcgcgacttcgtagtgatcgacggagcgccccaggcggcggacttggctgtgtccgcgatcaaggcagccg
acttcgtgctgattccggtgcagccaagcccttacgacatatgggccaccgccgacctggtggagctggttaagcagcgc
attgaggtcacggatggaaggctacaagcggcctttgtcgtgtcgcgggcgatcaaaggcacgcgcatcggcggtgaggt
tgccgaggcgctggccgggtacgagctgcccattcttgagtcccgtatcacgcagcgcgtgagctacccaggcactgccg
ccgccggcacaaccgttcttgaatcagaacccgagggcgacgctgcccgcgaggtccaggcgctggccgctgaaattaaa
tcaaaactcatttgagttaatgaggtaaagagaaaatgagcaaaagcacaaacacgctaagtgccggccgtccgagcgca
cgcagcagcaaggctgcaacgttggccagcctggcagacacgccagccatgaagcgggtcaactttcagttgccggcgga
ggatcacaccaagctgaagatgtacgcggtacgccaaggcaagaccattaccgagctgctatctgaatacatcgcgcagc
taccagagtaaatgagcaaatgaataaatgagtagatgaattttagcggctaaaggaggcggcatggaaaatcaagaaca
accaggcaccgacgccgtggaatgccccatgtgtggaggaacgggcggttggccaggcgtaagcggctgggttgtctgcc
ggccctgcaatggcactggaacccccaagcccgaggaatcggcgtgacggtcgcaaaccatccggcccggtacaaatcgg
cgcggcgctgggtgatgacctggtggagaagttgaaggccgcgcaggccgcccagcggcaacgcatcgaggcagaagcac
gccccggtgaatcgtggcaagcggccgctgatcgaatccgcaaagaatcccggcaaccgccggcagccggtgcgccgtcg
attaggaagccgcccaagggcgacgagcaaccagatttttcgttccgatgctctatgacgtgggcacccgcgatagtcg

cagcatcatggacgtggccgttttccgtctgtcgaagcgtgaccgacgagctggcgaggtgatccgctacgagcttccag
acgggcacgtagaggtttccgcagggccggccggcatggccagtgtgtgggattacgacctggtactgatggcggtttcc
catctaaccgaatccatgaaccgataccgggaagggaagggagacaagcccggccgcgtgttccgtccacacgttgcgga
cgtactcaagttctgccggcgagccgatggcggaaagcagaaagacgacctggtagaaacctgcattcggttaaacacca
cgcacgttgccatgcagcgtacgaagaaggccaagaacggccgcctggtgacggtatccgagggtgaagccttgattagc
cgctacaagatcgtaaagagcgaaaccgggcggccggagtacatcgagatcgagctagctgattggatgtaccgcgagat
cacagaaggcaagaacccggacgtgctgacggttcaccccgattactttttgatcgatcccggcatcggccgttttctct
accgcctggcacgccgcgccgcaggcaaggcagaagccagatggttgttcaagacgatctacgaacgcagtggcagcgcc
ggagagttcaagaagttctgtttcaccgtgcgcaagctgatcgggtcaaatgacctgccggagtacgatttgaaggagga
ggcggggcaggctggcccgatcctagtcatgcgctaccgcaacctgatcgagggcgaagcatccgccggttcctaatgta
cggagcagatgctagggcaaattgccctagcaggggaaaaaggtcgaaaaggtctctttcctgtggatagcacgtacatt
gggaacccaaagccgtacattgggaaccggaacccgtacattgggaacccaaagccgtacattgggaaccggtcacacat
gtaagtgactgatataaaagagaaaaaaggcgatttttccgcctaaaactctttaaaacttattaaaactcttaaaaccc
gcctggcctgtgcataactgtctggccagcgcacagccgaagagctgcaaaaagcgcctacccttcggtcgctgcgctcc
ctacgccccgccgcttcgcgtcggcctatcgcggccgctggccgctcaaaaatggctggcctacggccaggcaatctacc
agggcgcggacaagccgcgccgtcgccactcgaccgccggcgcccacatcaaggcaccctgcctcgcgcgtttcggtgat
gacggtgaaaacctctgacacatgcagctcccggagacggtcacagcttgtctgtaagcggatgccgggagcagacaagc
ccgtcagggcgcgtcagcgggtgttggcgggtgtcggggcgcagccatgacccagtcacgtagcgatagcggagtgtata
ctggcttaactatgcggcatcagagcagattgtactgagagtgcaccatatgcggtgtgaaataccgcacagatgcgtaa
ggagaaaataccgcatcaggcgctcttccgcttcctcgctcactgactcgctgcgctcggtcgttcggctgcggcgagcg
gtatcagctcactcaaaggcggtaatacggttatccacagaatcaggggataacgcaggaaagaacatgtgagcaaaagg
ccagcaaaaggccaggaaccgtaaaaaggccgcgttgctggcgtttttccataggctccgcccccctgacgagcatcaca
aaaatcgacgctcaagtcagaggtggcgaaacccgacaggactataaagataccaggcgtttccccctggaagctccctc
gtgcgctctcctgttccgaccctgccgcttaccggatacctgtccgcctttctcccttcgggaagcgtggcgctttctca
tagctcacgctgtaggtatctcagttcggtgtaggtcgttcgctccaagctgggctgtgtgcacgaaccccccgttcagc
ccgaccgctgcgccttatccggtaactatcgtcttgagtccaacccggtaagacacgacttatcgccactggcagcagcc
actggtaacaggattagcagagcgaggtatgtaggcggtgctacagagttcttgaagtggtggcctaactacggctacac
tagaaggacagtatttggtatctgcgctctgctgaagccagttaccttcggaaaaagagttggtagctcttgatccggca
aacaaaccaccgctggtagcggtggtttttttgtttgcaagcagcagattacgcgcagaaaaaaaggatctcaagaagat
cctttgatcttttctacggggtctgacgctcagtggaacgaaaactcacgttaagggattttggtcatgcatgatatatc
tcccaatttgtgtagggcttattatgcacgcttaaaaataataaaagcagacttgacctgatagtttggctgtgagcaat
tatgtgcttagtgcatctaatcgcttgagttaacgccggcgaagcggcgtcggcttgaacgaatttctagctagacatta
tttgccgactaccttggtgatctcgcctttcacgtagtggacaaattcttccaactgatctgcgcgcgaggccaagcgat
cttcttcttgtccaagataagcctgtctagcttcaagtatgacgggctgatactgggccggcaggcgctccattgcccag
tcggcagcgacatccttcggcgcgattttgccggttactgcgctgtaccaaatgcgggacaacgtaagcactacatttcg
ctcatcgccagcccagtcgggcggcgagttccatagcgttaaggtttcatttagcgcctcaaatagatcctgttcaggaa
ccggatcaaagagttcctccgccgctggacctaccaaggcaacgctatgttctcttgcttttgtcagcaagatagccaga
tcaatgtcgatcgtggctggctcgaagatacctgcaagaatgtcattgcgctgccattctccaaattgcagttcgcgctt
agctggataacgccacggaatgatgtcgtcgtgcacaacaatggtgacttctacagcgcggagaatctcgctctctccag
gggaagccgaagtttccaaaaggtcgttgatcaaagctcgccgcgttgtttcatcaagccttacggtcaccgtaaccagc
aaatcaatatcactgtgtggcttcaggccgccatccactgcggagccgtacaaatgtacggccagcaacgtcggttcgag
atggcgctcgatgacgccaactacctctgatagttgagtcgatacttcggcgatcaccgcttccccc atgatgtttaact
ttgttttagggcgactgccctgctgcgtaacatcgttgctgctccataacatcaaacatcgacccacggcgtaacgcgct
tgctgcttggatgcccgaggcatagactgtaccccaaaaaaacatgtcataacaagaagccatgaaaaccgccactgcgc
cgttaccaccgctgcgttcggtcaaggttctggaccagttgcgtgacggcagttacgctacttgcattacagcttacgaa
ccgaacgaggcttatgtccactgggttcgtgcccgaattgatcacaggcagcaacgctctgtcatcgttacaatcaacat
gctaccctccgcgagatcatccgtgtttcaaacccggcagcttagttgccgttcttccgaatagcatcggtaacatgagc
aaagtctgccgccttacaacggctctcccgctgacgccgtcccggactgatgggctgcctgtatcgagtggtgattttgt
gccgagctgccggtcggggagctgttggctggctggtggcaggatatattgtggtgtaaacaaattgacgcttagacaac
ttaataacacattgcggacgtttttaatgtactgaattaacgccgaattgaattatcagcttgcatgccggtcgatctag
taacatagatgacaccgCGCGgaagttcctatactttctagagaataggaacttcCTAGGaccgcgaattatcatacatg
agaattaagggagtcacgttatgacccccgccgatgacgcgggacaagccgttttacgtttggaactgacagaaccgcaa
cgttgaaggagccactcagccgcgggtttctggagtttaatgagctaagcacatacgtcagaaaccattattgcgcgttc
aaaagtcgcctaaggtcactatcagctagcaaatatttcttgtcaaaaatgctccactgacgttccataaattcccctcg
gtatccaattagagtctcatattcactctcaactcgatcgaggcatgattgaacaagatggattgcacgcaggttctccg
gccgcttgggtggagaggctattcggctatgactgggcacaacagacaatcggctgctctgatgccgccgtgttccggct
gtcagcgcaggggcgcccggttctttttgtcaagaccgacctgtccggtgccctgaatgaactccaagacgaggcagcgc

ggctatcgtggctggccacgacgggcgttccttgcgcagctgtgctcgacgttgtcactgaagcgggaagggactggctg
ctattgggcgaagtgccggggcaggatctcctgtcatctcaccttgctcctgccgagaaagtatccatcatggctgatgc
aatgcggcggctgcatacgcttgatccggctacctgcccattcgaccaccaagcgaaacatcgcatcgagcgaggacgta
ctcggatggaagccggtcttgtcgatcaggatgatctggacgaagagcatcaggggctcgcgccagccgaactgttcgcc
aggctcaaggcgcggatgcccgacggcgaggatctcgtcgtgacccagggcgatgcctgcttgccgaatatcatggtgga
aaatggccgcttttctggattcatcgactgtggccggctgggtgtggcggaccgctatcaggacatagcgttggctaccc
gtgatattgctgaagagcttggcggcgaatgggctgaccgcttcctcgtgctttacggtatcgccgctcccgattcgcag
cgcatcgccttctatcgccttcttgacgagttcttctgagcgggactctggggttcggactctagctagagtcaagcaga
tcgttcaaacatttggcaataaagtttcttaagattgaatcctgttgccggtcttgcgatgattatcatataatttctgt
tgaattacgttaagcatgtaataattaacatgtaatgcatgacgttatttatgagatgggtttttgattagagtcccgc
aattatacatttaatacgcgatagaaaacaaaatatagcgcgcaaactaggataaattatcgcgcgcggtgtcatctatg
ttactagatcgaccggcatgcaagctgatgaagcctgcaggagaattcgatcgccaagcttCtcgagagtcagcctttta
agagatagaatttaaaatataatttgcgtaaaacattattaaaaatacaaatttataaattaagttcaactcatcctatc
tcactctttaaatacgatgtttacttattagactcattaataaaaaaaaaaaaaatcatttgtacaaagcccaccataaa
ggcaatttgggcctggtagaccaatcctaaccaatgtctggttaagatggtccaatcccgaaacttctagttgcggttcg
aagaagtccagaatgtttctgaaagtttcagaaaattctagttttgagattttcagaagtacggcatgatgatgcataac
aaggactttctcgaaagtactatattgctcctctacatcattttaaataccccatgtgtcctttgaagacacatcacaga
aagaagtgaaggcatcgtatcaagcttacctgcggtgacgtctaagcatgccacaatttgatatattatgtaaaacacca
cctaaggtgcttgttcgtcagtttgtggaaaggtttgaaagaccttcaggtgagaaaatagcattatgtgctgctgaact
aacctatttatgttggatgattacacataacggaacagcaatcaagagagccacattcatgagctataatactatcataa
gcaattcgctgagtttcgatattgtcaataaatcactccagtttaaatacaagacgcaaaaagcaacaattctggaagcc
tcattaaagaaattgattcctgcttgggaatttacaattattccttactatggacaaaaacatcaatctgatatcactga
tattgtaagtagtttgcaattacagttcgaatcatcggaagaagcagataagggaaatagccacagtaaaaaaatgctta
aagcacttctaagtgagggtgaaagcatctgggagatcactgagaaaatactaaattcgtttgagtatacttcgagattt
acaaaaacaaaaactttataccaattcctcttcctagctactttcatcaattgtggaagattcagcgatattaagaacgt
tgatccgaaatcatttaaattagtccaaaataagtatctgggagtaataatccagtgtttagtgacagagacaaagacaa
gcgttagcaggtaagtttctgcttctacctttgatatatatataataattatcattaattagtagtaatataatatttca
aatattttttcaaaataaaagaatgtagtatatagcaattgcttttctgtagtttataagtgtgtatattttaatttat
aacttttctaatatatgaccaaaatttgttgatgtgcaggcacatatacttctttagcgcaaggggtaggatcgatccac
ttgtatatttggatgaatttttgaggaattctgaaccagtcctaaaacgagtaaataggaccggcaattcttcaagcaat
aaacaggaataccaattattaaaagataacttagtcagatcgtacaataaagctttgaagaaaaatgcgccttattcaat
ctttgctataaaaaatggcccaaaatctcacattggaagacatttgatgacctcatttctttcaatgaagggcctaacgg
agttgactaatgttgtgggaaattggagcgataagcgtgcttctgccgtggccaggacaacgtatactcatcagataaca
gcaatacctgatcactacttcgcactagtttctcggtactatgcatatgatccaatatcaaaggaaatgatagcattgaa
ggatgagactaatccaattgaggagtggcagcatatagaacagctaaagggtagtgctgaaggaagcatacgataccccg
catggaatgggataatatcacaggaggtactagactacctttcatcctacataaatagacgcatatgaggcgcgcgatcg
ttcaaacatttggcaataaagtttcttaagattgaatcctgttgccggtcttgcgatgattatcatataatttctgttga
attacgttaagcatgtaataattaacatgtaatgcatgacgttatttatgagatgggtttttatgattagagtcccgcaa
ttatacatttaatacgcgatagaaaacaaaatatagcgcgcaaactaggataaattatcgcgcgcggtgtcatctatgtt
actagatcgctcgtccggaggccatggcggccgccgctttccttacattctgagcctctttccttctaatccactcatct
gcatcttcttgtgtccttactaatacctcattggttccaaattccctccctttaagcaccagctcgtttctgttcttcca
cagcctcccaagtatccaagggactaaagcctccacattcttcagatcaggatattcttgtttaagatgttgaactctat
ggaggtttgtatgaactgatgatctaggaccggataagttcccttcttcatagcgaacttattcaaagaatgttttgtgt
atcattcttgttacattgttattaatgaaaaaatattattggtcattggactgaacacgagtgttaaatatggaccaggc
cccaaataagatccattgatatatgaattaaataacaagaataaatcgagtcaccaaaccacttgcctttttttaacgaga
cttgttcaccaacttgatacaaaagtcattatcctatgcaaatcaataatcatacaaaaatatccaataacactaaaaaa
ttaaaagaaatggataatttcacaatatgttatacgataaagaagttacttttccaagaaattcactgattttataagcc
cacttgcattagataaatggcaaaaaaaaacaaaaaggaaaagaaataaagcacgaagaattctagaaaatacgaaatac
gcttcaatgcagtgggacccacggttcaattattgccaattttcagctccaccgtatatttaaaaaataaaacgataatg
ctaaaaaaatataaatcgtaacgatcgttaaatctcaacggctggatcttatgacgaccgttagaaattgtggttgtcga
cgagtcagtaataaacggcgtcaaagtggttgcagccggcacacacgagtcgtgtttatcaactcaaagcacaaatactt
ttcctcaacctaaaaataaggcaattagccaaaaacaactttgcgtgtaaacaacgctcaatacacgtgtcattttatta
ttagctattgcttcaccgccttagctttctcgtgacctagtcgtcctcgtcttttcttcttcttcttctataaaacaata
cccaaagagctcttcttcttcacaattcagatttcaatttctcaaaatcttaaaaactttctctcaattctctctaccgt
gatcaaggtaaatttctgtgttccttattctctcaaaatcttcgattttgttttcgttcgatcccaatttcgtatatgtt
ctttggtttagattctgttaatcttagatcgaagacgattttctgggtttgatcgttagatatcatcttaattctcgatt
agggtttcatagatatcatccgatttgttcaaataatttgagttttgtcgaataattactcttcgatttgtgatttctat

ctagatctggtgttagtttctagtttgtgcgatcgaatttgtcgattaatctgagtttttctgattaacaggtactagtg
gatcctccataactatggcccccaagaagaagcgcaaagtgggaatccacggagctccagacaagaagtacagcattggt
ctggacatcgggaccaactccgtcggctgggccgttatcaccgatgagtacaaagtgccttctaagaagttcaaagtcct
cggaaacactgacaggcacagcatcaagaagaacttgattggcgcccttctcttcgactccggcgaaaccgctgaggcca
cccgtctgaagcgcaccgccagaaggcgctacacccgtcgcaagaataggatctgctacctccaggagatcttcagcaac
gagatggccaaagtggacgattccttctttcaccgtttggaggagagcttccttgtcgaggaggacaagaagcacgagcg
ccatcccatcttcggaaacattgttgacgaggtggcttaccacgagaagtatccaactatctaccacctcagaaagaagc
tggtggactccaccgacaaggccgatttgaggctcatctaccttgccctcgctcacatgatcaagttccgcggtcacttc
ctgattgagggcgacctcaaccccgacaactctgacgtcgataagttgttcatccaactggtccagacctacaaccagct
tttcgaggagaatcctatcaacgcctccggggttgacgccaaggctattctcagcgcccgtctgtccaagagccgcaggc
ttgagaacttgatcgcccagctcccaggagagaagaagaacggcctcttcggaaacctgatcgctctctccttgggcctt
accccctaacttcaagtctaattttgacctcgccgaggacgccaagctgcaactcagcaaggacacctacgatgacgactt
ggacaacctgctcgcccagatcggcgatcagtacgctgaccttttcttggccgccaagaacctctccgacgctattctgc
tctccgacatccttcgcgtgaacactgagatcaccaaggccccactgagcgcctccatgatcaagcgttacgacgaacac
caccaagatttgaccctcctcaaggctcttgtcagacagcagctgcccgagaagtacaaggagattttcttcgaccagag
caagaacggatacgccggttacatcgacggcggggcctcccaagaggagttctacaagttcatcaagcccatcttggaga
agatggacggcaccgaagagctgctcgtgaagctcaaccgcgaggatcttttgaggaagcagcgtaccttcgacaacgga
tctattccccaccagatccatctcggcgagctgcacgctatcctccgcaggcaagaggacttctatccattccttaagga
caatcgcgagaagattgaaaagatcctcacttttagaatcccttactacgtcggacccttggcccgtggcaacagccgct
tcgcctggatgaccaggaagtccgaggaaaccatcaccccatggaacttcgaggaggtggttgacaagggggccagcgct
cagtccttcattgagcgcatgaccaacttcgataagaacctgcccaacgagaaagtcctcccaaagcactcccttctgta
cgagtacttcactgtctacaatgagttgaccaaagtgaagtacgtgaccgagggtatgcgtaagcctgccttcctcagcg
gcgagcagaagaaggccatcgttgacctcctgttcaagaccaacaggaaagtcaccgtgaagcagcttaaggaggactac
ttcaagaagatcgagtgctttgactctgtcgagatcagcggagtggaggatcgcttcaacgcttccttgggcacttacca
cgacctcctgaagattatcaaggacaaggacttcctcgacaacgaggagaacgaagatatcttggaggacatcgttctta
ccctcaccctgttcgaggacagagagatgattgaggaacgcctcaagacctacgcccacttgttcgacgataaagtcatg
aagcaacttaagcgtaggcgctacaccggatggggcaggctgtcccgtaagctcatcaacggtatccgcgacaagcagag
cggcaagactattctcgacttcttgaagtccgacggattcgccaatcgcaacttcatgcagctgatccacgacgattctc
ttaccttcaaggaggacatccaaaaggctcaagtgtccggccagggcgacagcctccacgagcacatcgccaacctggcc
gggagccccgctattaagaagggaatcctccagaccgtcaaggtggttgacgagttggtcaaggtgatgggcagacataa
gccagagaacatcgtcatcgagatggccagggaaaaccagaccacccagaagggacagaagaactcccgtgagcgcatga
agaggattgaggagggcatcaaggagcttggttcccagatcctcaaggagcaccccgtggaaaacacccaactccagaat
gagaagctgtatttgtactaccttcagaacgggcgcgatatgtacgttgaccaagagctggacatcaaccgcctcagcga
ctacgacgtggatcacattgtccctcagtcctttctcaaggacgactctatcgacaacaaggtgttgactagaagcgata
agaaccgcggcaagtccgacaacgtcccatccgaggaggtggtcaagaagatgaagaattactggaggcagctgcttaac
gccaagctcatcacccagcgcaagttcgacaacttgaccaaggccgagcgtggaggcctcagcgaactggacaaggctgg
attcatcaagaggcaacttgttgaaacccgccagattaccaagcacgtggcccagatcctcgactcccgtatgaacacta
agtacgatgagaacgacaagctgatccgcgaggtcaaagtgattaccctcaagagcaagctcgtgtctgacttcagaaag
gacttccaattctacaaggttagggagatcaacaactaccaccacgcccacgatgcttaccttaatgccgtggtcggcac
cgccttgatcaagaagtaccccaagctggagtccgagttcgtgtatggtgactacaaggtctacgacgttcgcaagatga
tcgctaagagcgagcaggagattggcaaggccaccgccaagtacttcttctactccaacatcatgaacttctttaagacc
gagatcactttggctaacggggagatccgtaagcgccctctcattgaaaccaacggagaaaccggcgagatcgtgtggga
caagggcagggacttcgccaccgtcagaaaagtgctgagcatgccccaagtcaacatcgtgaagaaaaccgaggttcaga
ctggaggcttctccaaggagtctatcctcccaaagcgcaattccgataagttgattgcccgtaagaaggactgggacccc
aagaagtacggtggattcgacagcccaaccgtcgcctactccgtgcttgtcgtggctaaagttgagaagggcaagagcaa
gaagctcaagtccgtcaaggagctgctcgggatcaccatcatggagcgcagctccttcgagaagaaccctattgatttcc
ttgaggccaagggctacaaggaagtgaagaaggacttgatcatcaagctccccaagtactctctgttcgagcttgagaac
ggaaggaagcgtatgctcgcctccgctggcgagctgcaaaagggaaacgagttggccctcccaagcaagtacgtcaactt
cctgtacctcgcctcccactatgagaagctcaagggcagccccgaggacaacgaacagaagcagttgtttgtggagcagc
ataagcactaccttgacgagatcattgagcagatcagcgagttctccaagcgcgttatcctggctgacgccaatctcgat
aaagtcctttctgcctacaacaagcacagggacaagcctatcagagagcaggctgaaaacattatccacttgttcaccct
caccaacctgggtgccccagccgccttcaagtacttcgacactaccatcgaccgcaagcgttacacctccaccaaggaag
tgctcgatgctacccttatccaccagagcattactgggttgtacgaaaccaggatcgacctgtcccaactcggcggagac
aagcgccccgccgccaccaagaaggctggccaggccaagaagaagaagtaacccgggatcgttcaaacatttggcaataa
agtttcttaagattgaatcctgttgccggtcttgcgatgattatcatataatttctgttgaattacgttaagcatgtaat
aattaacatgtaatgcatgacgttatttatgagatgggtttttatgattagagtcccgcaattatacatttaatacgcga
tagaaaacaaaatatagcgcgcaaactaggataaattatcgcgcgcggtgtcatctatgttactagatcgctcgaattcc

ttcgttgaacaacggaaactcgacttgccttccgcacaatacatcatttcttcttagctttttttcttcttcttcgttca
tacagtttttttttgtttatcagcttacattttcttgaaccgtagctttcgttttcttctttttaactttccattcggag
tttttgtatcttgtttcatagtttgtcccaggattagaatgattaggcatcgaaccttcaagaatttgattgaataaaac
atcttcattcttaagatatgaagataatcttcaaaaggcccctgggaatctgaaagaagagaagcaggcccatttatatg
ggaaagaacaatagtatttcttatataggcccatttaagttgaaaacaatcttcaaaagtcccacatcgcttagataaga
aaacgaagctgagtttatatacagctagagtcgaagtagtgattGGTACTCTATCCCTTATGTAgttttagagctagaaa
tagcaagttaaaataaggctagtccgttatcaacttgaaaaagtggcaccgagtcggtgcttttttgtcgacaggcctt
aagggccagatcttgggcccgaagttcctatactttctagagaataggaacttccgcacatatcgaggtggacattacct
acgccccggtacc

Supplementary Figure 5. Pictures of the two types of regenerations used in this work. Upper part: protoplast regeneration used in transient delivery (Methods 1 and 2). Lower part: regeneration from leaf explants used in stable delivery (Method 3).

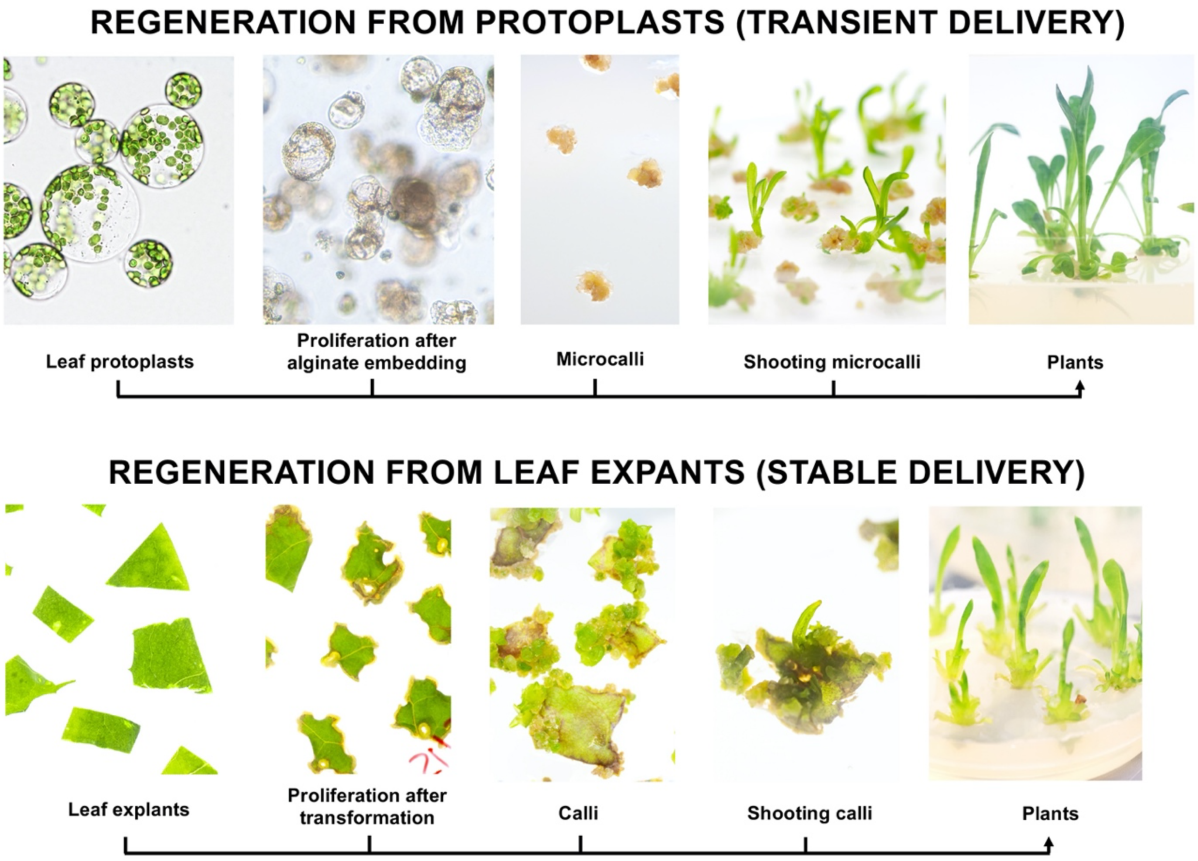

Supplementary Figure 6. Alignment of CiGAS genes (whole exon 4) where the Common Guide RNA was designed. PAM sequence is highlighted in yellow, and the sgRNA sequence is in red. The single mismatches of CiGAS-S3 and CiGAS-L are in bold. The sequence of the DDxxD motif is depicted in blue.

```
CiGAS-S1-Ex4        GTGGTGGAAGGATATGAGGTTCCAGGAAACTACTCCTTACATAAGGGATAGAGTACCAGA
CiGAS-S2-Ex4        GTGGTGGAAGGATATGAGGTTCCAGGAAACTACTCCTTACATAAGGGATAGAGTACCAGA
CiGAS-S3-Ex4        GTGGTGGAAGGATATGAGGTTCCAAGAAACTACTCCTTATATAAGGGATAGAGTACCCGA
CiGAS-L-Ex4         GTGGTGGAAAGATATGCAATTCCAACAATCCGTCCCTTACATAAGAGATAGAGTACCGGA
                    ********* ******   *****  ** *    ***** ***** *********** **

CiGAS-S1-Ex4        GATTTACTTATGGATATTGGGATTGTACTTTGAGCCTCGTTACTCCTTGGCACGAATCAT
CiGAS-S2-Ex4        GATTTACTTATGGATATTGGGATTGTACTTTGAGCCTCGTTACTCCTTGGCACGAATCAT
```

```
CiGAS-S3-Ex4      GATCTACTTATGGATATTAGGATTGTACTTTGAGCCTCGTTACTCCTTGGCACGAATCAT
CiGAS-L-Ex4       GATATACCTATGGATTTTGGGGTTATATTTCGAGCCGTATTACTCTCGGGCACGTATCAT
                  *** *** ******* ** ** ** ** ** *****   ******   ****** *****

CiGAS-S1-Ex4      CGCCACAAAAATAACATTGTTTCTGGTGGTGCTAGATGACACATATGATGCGTACGCTAC
CiGAS-S2-Ex4      CGCCACAAAAATTACATTGTTCCTGGTGGTTCTAGATGACACATATGATGCATACGCTAC
CiGAS-S3-Ex4      CGCCACAAAAATTACATTGTTTTTGGTGGTGCTAGACGACACATATGATGCATATGCTAC
CiGAS-L-Ex4       AGCCACTAAAATCACGTTGTTCTTGGTGGTTTTGGACGATACATATGACGCGTATGCTAC
                   ***** ***** ** *****  *******  * ** ** ******** ** ** *****

CiGAS-S1-Ex4      CATTGAAGAGATTCGACTTCTAACAGATGCCATAAACAG
CiGAS-S2-Ex4      CATTGAAGAGATTCGACTTCTGACAGATGCCATAAACAG
CiGAS-S3-Ex4      AATTGAAGAAATTCGTCTTTTAACTGATGCCATAAATAG
CiGAS-L-Ex4       AATTGACGAGATCCGATCGATCACAGATGCGATTAATAG
                   ***** ** ** **     * ** ***** ** ** **
```

Supplementary Figure 7. Number of reads and respective percentage on the total for the protoplast transient assay with RNPs. PAM is highlighted, the target sequence is in uppercase and insertion are in bold.

CiGAS-S1

aaactactcctTAC-ATAAGGGATAGAGTACCaga	WT	3304	47.5%
aaactactcctTAC----AGGGATAGAGTACCaga	-3	868	12.5%
aaactactcctTA------------GAGTACCaga	-11	760	10.9%
aaactactcctTA-----------AGAGTACCaga	-10	576	8.3%
aaactactcctTAC---AAGGGATAGAGTACCaga	-2	536	7.7%
aaactactcctTAC-**A**ATAAGGGATAGAGTACCag	+1	481	6.9%
aaactactcctTAC-----GGGATAGAGTACCaga	-4	434	6.2%

CiGAS-S2

aaactactcctTAC-ATAAGGGATAGAGTACCaga	WT	4021	45.9%
aaactactcctTA----------TAGAGTACCaga	-9	872	9.9%
aaactactcctTA---------------GTACCaga	-13	867	9.9%
aaactactcctTAC----AGGGATAGAGTACCaga	-3	850	9.7%
aaactactcctTAC--------ATAGAGTACCaga	-7	664	7.6%
aaactactcctTAC----------AGAGTACCaga	-9	570	6.5%
aaactactcctTAC-**A**ATAAGGGATAGAGTACCag	+1	474	5.4%
aaactactcctTAC---AAGGGATAGAGTACCaga	-2	431	4.9%
